# Gum Arabic: A Commodity with Versatile Formulations and Applications

**DOI:** 10.3390/nano15040290

**Published:** 2025-02-13

**Authors:** Shaymaa A. Mohamed, Asmaa M. Elsherbini, Heba R. Alrefaey, Kareem Adelrahman, Alshaimaa Moustafa, Nishal M. Egodawaththa, Kaitlyn E. Crawford, Nasri Nesnas, Sally A. Sabra

**Affiliations:** 1 Department of Biotechnology, Institute of Graduate Studies and Research, Alexandria University, Alexandria 21526, Egypt; shaymaaabdelrhman@alexu.edu.eg (S.A.M.); asmaaelsherbiny33@gmail.com (A.M.E.); 2 Department of Chemistry and Chemical Engineering, Florida Institute of Technology, Melbourne, FL 32901, USA; he353346@ucf.edu (H.R.A.); enishalmaduj2020@my.fit.edu (N.M.E.); 3 Department of Materials Science and Engineering, University of Central Florida, Orlando, FL 32826, USA; kareem.abdelrahman@ucf.edu (K.A.); kcrawford@ucf.edu (K.E.C.); 4 Department of Materials Science, Institute of Graduate Studies and Research, Alexandria University, Alexandria 21526, Egypt; igsr.alshaimaa.samy@alexu.edu.eg; 5 Biionix Cluster, University of Central Florida, Orlando, FL 32827, USA; 6 Department of Chemistry, University of Central Florida, Orlando, FL 32816, USA; 7 NanoScience Technology Center, University of Central Florida, Orlando, FL 32826, USA

**Keywords:** Gum Arabic, nanoparticles, hydrogels, nanofibers, membranes, scaffolds, films

## Abstract

Gum Arabic (GA), or acacia gum, refers to the dried exudate produced by certain Acacia trees. GA is composed mainly of a mixture of polysaccharides and glycoproteins, with proportions that can slightly differ from one species to another. It is commonly utilized in the food and pharmaceutical industries as a stabilizer or an emulsifier owing to its biocompatibility, hydrophilicity, and antibacterial properties. In addition, GA can be manipulated as it possesses many functional groups that can be used in grafting, cross-linking, or chemical modifications to add a new feature to the developed material. In this review, we highlight recent GA-based formulations, including nanoparticles, hydrogels, nanofibers, membranes, or scaffolds, and their possible applications in tissue regeneration, cancer therapy, wound healing, biosensing, bioimaging, food packaging, and antimicrobial and antifouling membranes.

## 1. Gum Arabic (An Overview): Composition, Analogues, and Properties

**Figure 1:**
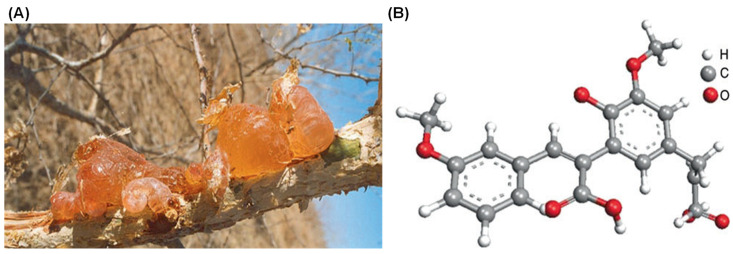
Gum Arabic formed on a wounded branch of *A. Senegal* (**A**) and chemical structure of the Acacia Gum with a backbone composed of 1,3-linked β-D-galactopyranosyl units and the side chains comprised of two to five 1,3-linked β-D-galactopyranosyl units, joined to the main chain by 1,6-linkages (**B**) [2].

**Figure 2:**
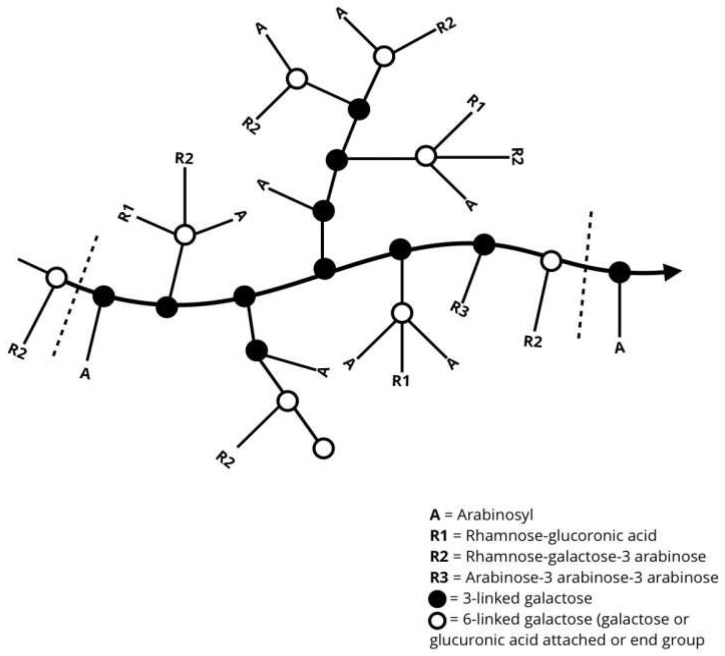
General representation of the molecular chain arrangement of gum Arabic [3].

Gum Arabic (GA), also known as gum acacia, is a naturally occurring gum made from the hardened sap of various species of the acacia tree, primarily *Acacia seyal* and *Acacia senegal* (Figure 1). GA has been utilized for thousands of years, serving as an essential material in various applications due to its unique properties. The chemical structure, composition, and analogues of GA play significant roles in determining its functionality and applications [1,2]. GA is primarily composed of a mixture of polysaccharides and glycoproteins, with the polysaccharide portion accounting for the majority of its mass. (Figure 2) [3].

**Figure 3:**
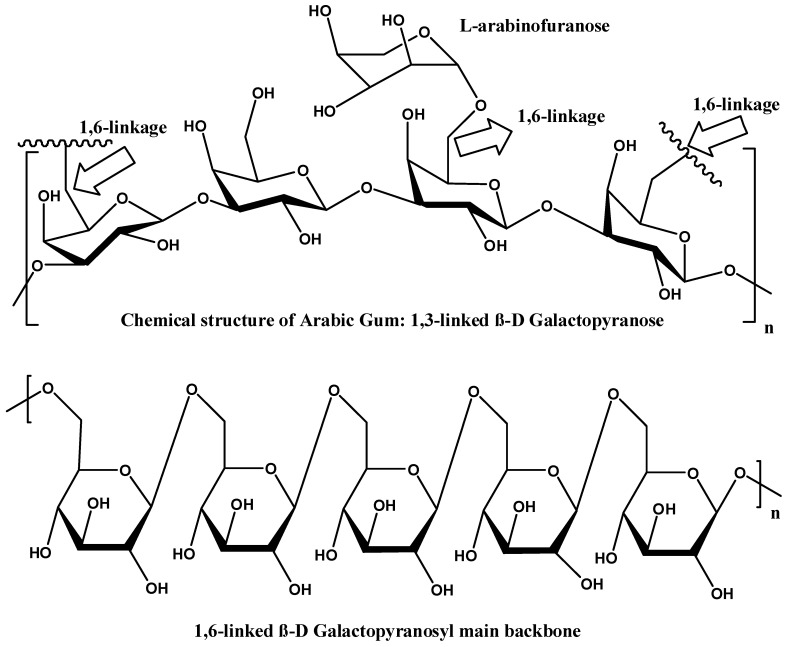
Chemical structure of 1,3-linked and 1,6 linked gum Arabic [4].

The polysaccharide portion of GA is primarily composed of (1→3)-linked β-D-galactopyranosyl units. The gums’ backbone is further branched at the O-6 position by side chains of various lengths (Figure 3). The side chains are primarily composed of α-L-arabinofuranosyl and β-D-galactopyranosyl units. The side chains can also include other sugars, such as L-arabinose and D-glucuronic acid, which contribute to the overall charge and solubility of the gum in aqueous solutions. Meanwhile, the presence of L-rhamnose at the terminal ends might influence the gum’s solubility and viscosity. The diverse saccharides in the structure of GA contribute to its anionic nature [4,5,6].

The protein part consists mainly of hydroxyproline, serine, and threonine residues, which are glycosylated with arabinogalactan, forming a complex network. Hydroxyproline is essential for the emulsifying properties of GA, with glycosylation enhancing the water solubility and functional properties [7]. This fraction typically comprises around 1–2% of the total weight of the gum. The molecular weight of GA varies significantly, ranging from approximately 250,000 to over 1,000,000 Daltons, depending on the source and processing methods [4,5,6]. The polydispersion index (PDI) of GA typically ranges from 1.5 to 2.5, which reflects its heterogeneous nature and broad distribution of molecular weights [4,5,6,7,8,9].

The exact chemical composition of GA can vary slightly depending on factors such as its botanical origin, harvest season, climate, tree age, and the conditions of the processing [10]. Studies have highlighted the variations between the chemical compositions of GA derived from *Acacia senegal* and *Acacia seyal*. One important study by Lopez-Torrez et al. confirmed that while both Acacia gums contain the same amino acids, the protein content is higher in *A. senegal* (2.7%) compared to *A. seyal* (1.0%). Key amino acids such as serine, leucine, proline, and hydroxyproline are the most abundant, making up more than 55% of the total amino acids in each type [11]. These findings are consistent with previous research on Acacia gums from various origins, which consistently show that *Acacia senegal* contains roughly double the amount of protein compared to *Acacia seyal*. Overall, the chemical properties of GA are affected by a combination of genetic and environmental factors, which contribute to its variability and functionality in different applications (Table 1) [2,10].

Hydrolyzed GA yields three main fractions, including glycoprotein, arabinogalactan, and arabinogalactan protein, each with a distinct molecular weight and chemical composition. The arabinogalactan portion constitutes about 88% of GA’s total weight, with a relatively low molecular weight (~300 kDa) and minimal protein content of less than 1%. The arabinogalactan protein portion accounts for approximately 10% of the gum and boasts a high molecular weight (~1500 kDa), with a protein content of around 10%. The glycoprotein fraction, representing less than 2% of the gum, has the lowest molecular weight but the highest protein content, ranging from 20% to 50%. Arabinogalactan protein is the most active component, and the main component responsible for the emulsifying properties of GA. It adsorbs onto the oil–water interface, forming a viscoelastic film that reduces interfacial tension due to its amphiphilic nature, which arises from the combination of hydrophobic protein chains and hydrophilic polysaccharide fragments [4,5].

Additionally, GA is biodegradable and environmentally friendly, making it suitable for applications with environmental concerns. It can be used in oil recovery as it exhibits non-Newtonian, shear-thinning behavior, causing a decrease in its viscosity with increasing shear rate, allowing for optimized flow properties in fluid dynamics-critical processes. The protein component of GA adsorbs onto the oil–water interface, and the polysaccharide part provides steric stabilization, preventing the coalescence of oil droplets. It maintains good thermal stability up to moderate temperatures, ensuring consistent performance within the typical operational range (30 °C to 70 °C) used in oil recovery processes (Table 2) [6,7,8,9,10].

Several analogues of GA have been identified and utilized in various applications, sharing similar properties but differing in specific compositions and structural characteristics. Ghatti gum, derived from *Anogeissus latifolia*, is similar to GA in its emulsifying properties but has a different monosaccharide composition, including higher proportions of uronic acids. Tragacanth gum, sourced from the *Astragalus* species, forms highly viscous solutions and gels with a higher ratio of galacturonic acid, and is often used in pharmaceuticals and food products for thickening properties. Karaya gum, obtained from *Sterculia* species, is used as a thickening agent with a unique structure rich in galactose and rhamnose units, and is commonly utilized in dental adhesives and as a bulk-forming laxative. Guar gum, from *Cyamopsis tetragonoloba* seeds, is primarily a galactomannan with a high viscosity profile, and is widely used in food applications as a stabilizer and thickener. Locust bean gum, derived from *Ceratonia siliqua* seeds, is another galactomannan used for its gelling properties, especially in combination with other hydrocolloids like xanthan gum to enhance gel strength and elasticity [8,9].

**Figure 4:**
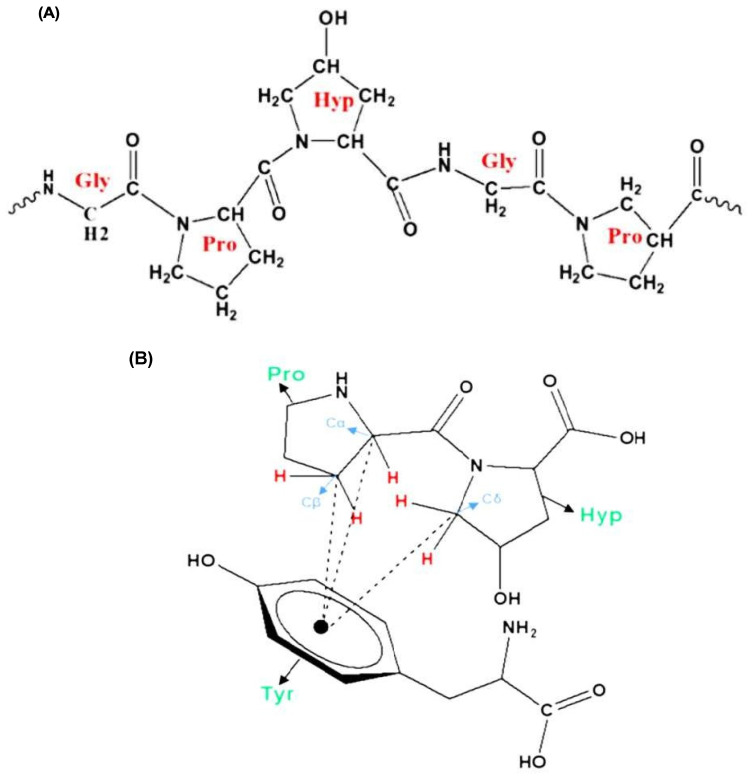
PPII helix with transamide bonds (**A**), CH...π interactions between two amino acids (Pro: proline and Hyp: hydroxyproline) and tyrosine (Tyr) amino acid (**B**) [17].

The amino acid composition of GA is the key factor determining its emulsifying properties. The most abundant amino acids in GA are hydroxyproline, followed by serine, proline, threonine, and many other amino acids with minor amounts. Owing to the conformational properties of proline and hydroxyproline, they can form polyproline II helix (PPII), which is a common conformation in the structure of proline-rich regions in proteins such as GA [11,12]. A PPII helix is a type of protein secondary structure occurring in proteins with repeated proline and/or hydroxyproline residues [13] (Figure 4A), and is thought to play a vital role in protein–protein [14] and protein–nucleic acid interactions [15]. Moreover, this helix has a relatively open extended structure with almost no internal hydrogen bonding, in contrast to more common helical secondary structures such as the α- and β-helices [16]. A structural analysis of GA proved that the aggregation of polyproline helices in GA, through several CH...π interactions (Figure 4B), is the primary driving force in the emulsification mechanism of GA. The π aromatic system is found in aromatic amino acids such as tyrosine and phenylalanine found in the structure of GA. These amino acids have a great affinity for hydrophobic molecules, and hence, the addition of GA to an oil/water system will cause the amino acids involved in the hydrophobic CH...π interactions to be naturally attracted to the hydrophobic oil phase, whereas the hydrophilic polysaccharide portion of GA will be directed towards the water phase, leading to the formation of stable emulsions [17].

The ratio between imino acids and aromatic amino acids is another important feature contributing to the emulsifying properties of GA. Proline and hydroxyproline (imino acids) amino acids in GA make approximately 300 residues, while the aromatic amino acids tyrosine and phenylalanine make approximately 50 residues. The low concentration of aromatic amino acids compared to imino acids suggests that all the aromatic amino acids are involved in the favorable CH...π interactions [17].

The complex coacervation technique is a common encapsulation procedure used to encapsulate active ingredients, and it includes two oppositely charged biopolymers. The encapsulation of hydrophobic active compounds requires the formation of an emulsion with one of the polymers, while the second polymer is utilized to enhance complex formation [18]. GA is one among the biopolymers that can be used as a shell material for complex coacervation [19,20]. It is negatively charged for all pH ranges, while proteins can be positively charged (below its isoelectric point) or negatively charged (above its isoelectric point) [21].

This technique depends on many factors, including the ionic strength, ratio of biopolymers and their concentration, pH, biopolymer composition, and conformational changes in secondary and tertiary structures [22]. Moreover, the potential of GA to form complex coacervation with soluble pea proteins was examined using GA from *Acacia senegal* and *Acacia seyal*, which differ in their composition and the amount of protein in their structure. GA from *Acacia senegal* was more efficient in binding to soluble pea proteins in comparison to GA from *Acacia seyal*. This higher affinity could be attributed to the presence of more glucuronic acids and a higher negative charge per molecule in GA from *Acacia Senegal*, giving more sites a chance to interact with positively charged proteins at an acidic pH (3.5–4), and hence forming more stable coacervates [23].

The main aim of this review is to highlight the most recent advances in GA-based formulations, including nanoparticles, hydrogels, nanofibers, membranes, or scaffolds, and their possible medical, pharmaceutical, or environmental applications.

## 2. Bibliometric Analysis

Bibliometric analysis is a quantitative approach to evaluating research trends, mapping knowledge domains, and identifying gaps in the scientific literature [24]. To systematically assess the scholarly landscape of Gum Arabic (GA), we conducted a bibliometric analysis using data extracted from the Scopus database, selected for its comprehensive coverage of peer-reviewed journals across disciplines. This section examines publication trends, citation patterns, keyword evolution, and collaboration networks to highlight research priorities and underexplored areas in GA studies.

The Scopus query (Table 3) was designed to retrieve articles published between January 2000 and July 2024, with data downloaded on 15 July 2024. To ensure relevance and quality, we restricted the search to final-stage journal articles in English. The search terms included “Gum Arabic” OR “Acacia Gum” OR “*Acacia senegal*” (accounting for taxonomic and regional naming variations).

The initial results identified 7708 documents when searching for GA-related keywords in titles, abstracts, or author keywords. After applying filters for document type (article), source type (journal), language (English), and publication stage (final), the dataset was refined to 5537 articles. The exclusion of 2171 documents resulted from the following criteria:

Document type: non-article publications (e.g., conference proceedings, book chapters, reviews, letters, editorials, and short surveys).

Language: non-English publications (e.g., studies published in Chinese, French, or other languages).

Source type: non-journal sources (e.g., trade magazines, preprint repositories, or non-academic databases).

Publication stage: pre-publication drafts, in-press articles, or non-final versions.

**Figure 5:**
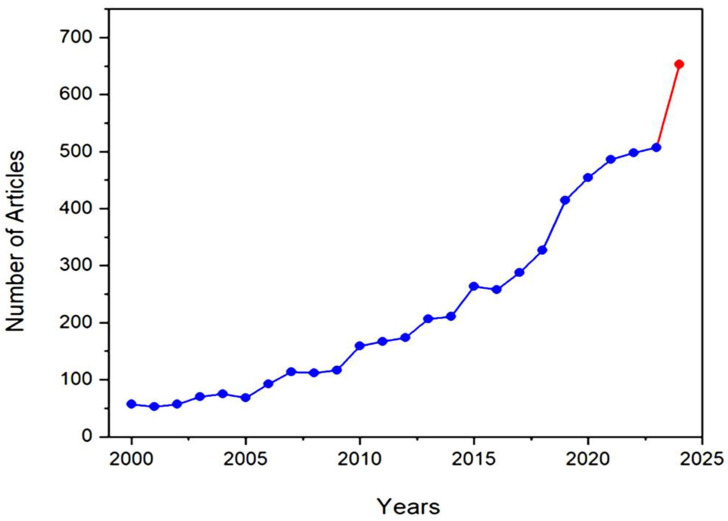
The number of articles that discuss Gum Arabic and its applications between 2000 and 2024. The red color indicates a sharp increase in the number of publications between 2023 and 2024.

One of the revealing bibliometric aspects is the average productivity per active year (PAY), which can be calculated by dividing the sum of the publications by the number of their active production years [24]. Figure 5 shows the progress of literature production over the years from 2000 until 15 July 2024, which shows a gradual increase in the productivity per active year (PAY), with significant growth during the last decade. To explain, PAY has significantly increased since 2015 to almost 385 publications per year, compared to less than 116 publications per year during the period of 2000–2014. The number of publications that discuss GA applications is expected to exceed 650 publications by the end of 2024, compared to nearly 500 publications in 2023. This increase in the number of publications reflects researchers’ growing interest in GA and its applications, as evidenced by the significant results highlighted in the literature.

**Figure 6:**
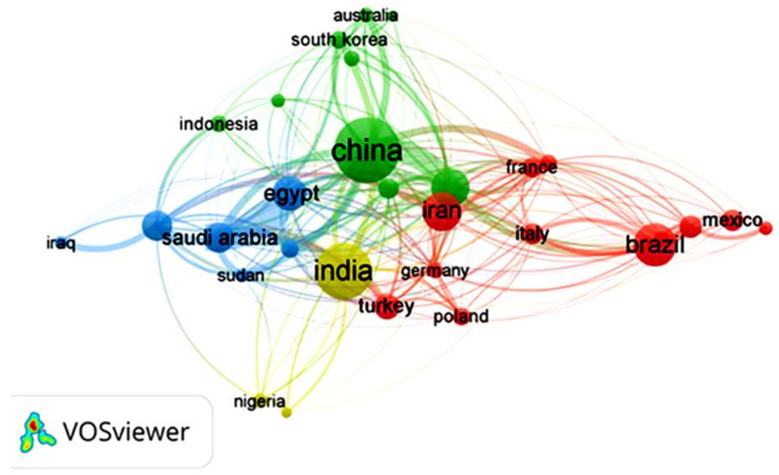
VOSviewer science mapping listing the top 30 countries that have produced research on Gum Arabic during the last decade and their co-authorship. The data was downloaded in .csv format for analysis and science mapping by VOSviewer 1.6.2 software.

Focusing on the last decade of research on GA material, it is observed that countries like China, India, Brazil, Iran, the United States, and Egypt are the highest contributors to scientific research on GA and its applications, as shown in Figure 6. One of the main reasons for researchers’ interest in studying GA in these countries, besides its remarkable properties, is that GA is produced naturally in these countries, and that some of them are of the main exporters of GA worldwide, such as China and India. Figure 6 shows the co-authorship between researchers from different countries, indicating research collaborations and cooperative research projects involving researchers and countries with a mutual interest in GA. Egypt, Saudi Arabia, India, China, and the United States have collaborated the most on co-authored publications during the last decade.

For instance, in a recent study by Hamza et al. [25], researchers from Egypt, China, Saudi Arabia, and France collaborated to design a new bio-based composite of GA and chitosan to act as a sorbent for metal decontamination from water. The study revealed that the bio-based composite was able to successfully decontaminate water samples collected from five wells located in Sinai (Egypt) with a uranium removal approaching 100% and the ability to recover metal ions like Cu(II), Fe(III), Zn(II) and Pb(II). Additionally, the bio-based composite exhibited antimicrobial activity against several pathogens, such as *S. aureus, B. subtilis*, and *E. coli*. In another study by Naushad et al. [26], in a collaboration between Saudi Arabian, Chinese, and Indian researchers, a novel GA nanocomposite hydrogel was designed to exhibit a photocatalytic performance that initiates the degradation of toxic dyes in water. The authors stated that the nanocomposite hydrogel degraded an average of 75% of the toxic dye under optimized conditions, demonstrating an important effort to protect the environment and human health.

**Figure 7:**
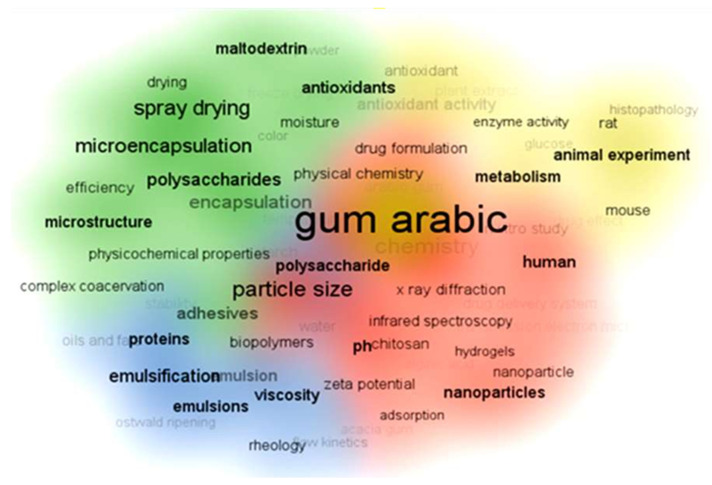
VOSviewer science mapping listing the top 100 keywords used in Gum Arabic articles during the last decade and their classifications. In recent Gum Arabic articles, the top 100 keywords are categorized into four clusters based on the articles’ focus, as shown in Figure. The red cluster pertains to the formulation of nanoparticles and nanofibers with keywords like polymer, hydrogels, nanoparticles, and chitosan. Methods for characterizing polymer composites, nanoparticles, and hydrogels include Transmission Electron Microscopy (TEM), X-ray diffraction (XRD), and particle size analysis. The blue cluster focuses on emulsions and stabilizing proteins, oils, and fats. For this category, it is suitable to characterize the products using flow kinetics, viscosity, and rheology concepts. Meanwhile, the green and yellow clusters address pharmaceutical, medical, and biological applications mentioning keywords like microencapsulation, drug delivery, animal tissue, in vitro studies, histopathology, and plant extracts. The data was downloaded in .csv format for analysis and science mapping by VOSviewer 1.6.2 software.

In recent GA articles, the top 100 keywords are categorized into four clusters based on the articles’ focus, as shown in Figure 7. The red cluster pertains to the formulation of nanoparticles and nanofibers with keywords like polymer, hydrogels, nanoparticles, and chitosan. Methods for characterizing polymer composites, nanoparticles, and hydrogels include Transmission Electron Microscopy (TEM), X-ray diffraction (XRD), and particle size analysis. The blue cluster focuses on emulsions and stabilizing proteins, oils, and fats. For this category, it is suitable to characterize the products using flow kinetics, viscosity, and rheology concepts. Meanwhile, the green and yellow clusters address pharmaceutical, medical, and biological applications, mentioning keywords like microencapsulation, drug delivery, animal tissue, in vitro studies, histopathology, and plant extracts.

**Figure 8:**
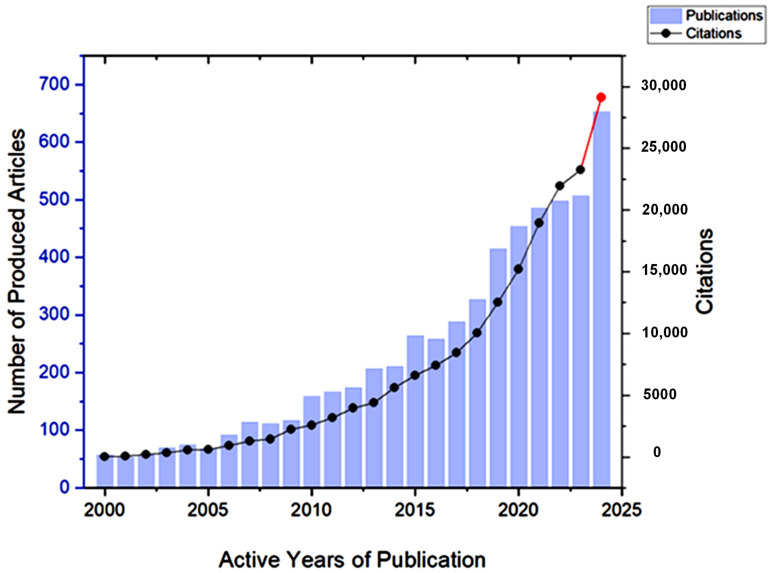
The development of the number of citations received by articles on Gum Arabic between 2000 and 2024. The red color indicates a sharp increase in the number of publications between 2023 and 2024.

Citation is an important aspect to consider when conducting bibliometrics on any research topic, as it indicates the level of interest from the scientific community in this topic [24]. The screened articles that discuss GA and its applications received 167,627 citations between 2000 and 2024, with the average number of citations per document exceeding 30. It is evident in Figure 8 that the number of citations has drastically increased during the past five years, which indicates the growing interest in Gum Arabic.

Many of the highly cited studies have provided opportunities for new formulation methods and applications of GA in various fields. For instance, Rad et al. [27] incorporated GA with poly (ε-caprolactone) (PCL) and zein to create a new nanocomposite scaffold by electrospinning; this showed promise as a biomaterial for skin regeneration. An in vitro trial of the multilayer scaffold showed significant mechanical and physical properties and the gradual release behavior of the loaded drug. Meanwhile, Amalraj et al. [28] used a GA polymer in a biocomposite with polyvinyl alcohol (PVA) and chitosan to prepare a polymer film that can be considered a promising alternative to wound dressing materials. The prepared film showed improved heat stability and significant mechanical properties, and exhibited antimicrobial characteristics.

Regarding the food packaging field, Kang et al. [29] developed a novel bio-nanocomposite film based on a GA polymer and reinforced it with cellulose nanocrystal (CNC) to be used for fruit packaging. The authors stated that incorporating 4 wt. % of CNC improved many of the GA nanocomposite’s characteristics, such as its tensile strength, elongation at break, water vapor and oxygen permeability, ultraviolet light barrier, and thermal stability, which was reflected in the delayed deterioration of fruits during storage. On the other hand, Wu et al. [30] explored the GA hydrogel for clean energy storage application as a biodegradable polymer electrolyte in aqueous zinc-ion batteries. They found that the GA hydrogel electrolyte protected the zinc anode from water-induced issues, such as corrosion and hydrogen evolution reactions. This was achieved by creating a hydrogen bond network between GA and water, which reduces the electrochemical activity of water.

## 3. Gum Arabic-Based Nanoparticles

**Figure 9:**
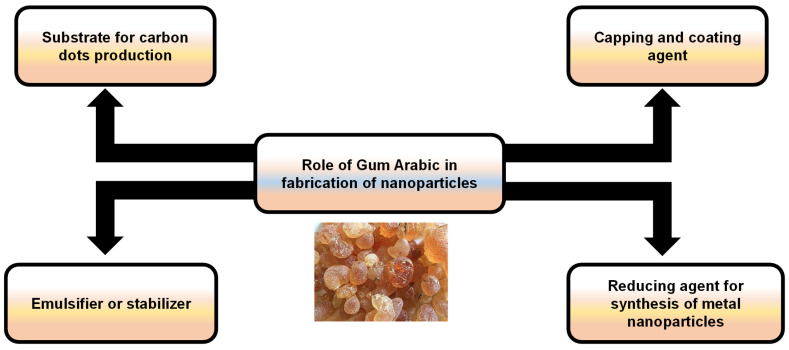
An illustration showing the possible roles of Gum Arabic in the fabrication of nanoparticles.

The good dispersion of NPs is a critical issue for their appropriate applicability, including drug delivery, biosensing, food additives, biomedical imaging, and therapy [31]. GA has excellent stabilizing properties, thereby preserving the colloidal stability of NPs by stimulating repulsive forces among particles, limiting their tendency to agglomerate [32]. Furthermore, the presence of a GA coating provides a protective layer that can improve their biocompatibility and control their release properties as a nontoxic, biodegradable natural component [33]. As a result, utilizing GA-based NPs offers great promise for enhancing the efficacy of bioactive substances (Figure 9).

### 3.1. Applications of GA-Based NPs

#### 3.1.1. Antimicrobial Activity

To manage dental caries induced by the oral pathogen *S. mutans*, GA can be employed in the bio-reduction of silver nitrate into AgNPs. The biosynthesized NPs were spherical, with a particle size less than 10 nm. GA-AgNPs effectively suppressed the growth of cariogenic bacteria with a MIC value of 10 µg/mL. In addition, the developed NPs inhibited the expression of virulence-associated glucosyl transference genes (gtfC gtfD and gtfB), which are responsible for the formation of dental pathogen biofilms on tooth surfaces [34].

In a similar approach, the antimicrobial efficacy of four commercial toothpastes (TPs) was evaluated for their ability to inhibit dental pathogens. Then, the TP displaying the lowest antimicrobial activity (31.3 µg/mL) was combined with greenly synthesized GA-AgNPs. The bio-synthesized nanosilver, with an average particle size of 220 nm, maintained its antimicrobial properties after adding TP. Furthermore, both GA-AgNPs combined with TP and GA-AgNPs alone exhibited cytotoxic effects on buccal mucosa fibroblast (BMF) cells, with GA-AgNP TP being found to be less toxic than bare AgNPs, thereby supporting their potential use as an anticariogenic agent [35].

For intracanal therapy, a GA nanocarrier was prepared by utilizing chitosan as a stabilizing agent and loaded with calcium hydroxide (Ca (OH)_2_) to evaluate their efficacy against *E. faecalis* biofilms. The developed Ca (OH)_2_-loaded GA-NPs had an oval shape, with a predominant Ca (OH)_2_ presence on the outer surface and a particle size of 60.47 nm. The designed nanocarrier demonstrated an improved anti-*E. faecalis* biofilm efficacy, as indicated by the decreased expression level of biofilm-associated genes (*ace*, *agg*, and *efaA*) and the reduced CFU count in *E. faecalis*-infected root canals compared to other treatment groups [36].

To conquer osteomyelitis caused by *E. coli*, mesoporous silica nanoparticles (MSNs) were fabricated using the modified Stöber method and loaded with moxifloxacin (MF). Subsequently, the nanovehicle was functionalized with GA and colistin (CN), yielding GA+CN-coated MF-loaded MSNs. Gum Arabic was selected as a carbon source utilized by bacterial enzymes, thereby promoting the retention of MSNs on the biofilm while serving as a polymeric mesh to enhance CN adsorption on MSN surfaces. Additionally, the nanosystem demonstrated non-cytotoxic and minimal hepatotoxicity, possibly due to the nature of MF and CN. In vitro evaluations revealed the effective inhibition of *E. coli* biofilm on bovine trabecular bone at 62.5 µg/mL. In an in vivo rabbit osteomyelitis model, the nanocomposite-treated group could eliminate more than 90% of the bacterial burden within infected bone [37].

Nanofluids of ZnO were greenly synthesized using NaOH as a reducing agent and GA as a stabilizer through a precipitation procedure assisted by a microwave heating process (450 W) at pH 10.0. The stabilization of nanofluid was ascribed to the protective role of GA, which prevents agglomeration via the steric effect, ensuring good storage stability for up to six months. Furthermore, stabilized nanofluid particles displayed a size distribution ranging from 200 to 350 nm, as opposed to the large diameter of 1020 nm seen in unstabilized nanofluids. ZnO nanofluids stabilized with 1.5% GA demonstrated enhanced antibacterial activity, as evidenced by a larger zone of inhibition compared to uncoated ZnO nanofluids [38].

In another investigation, copper nanoparticles (CuNPs) were synthesized using GA and L-ascorbic acid as capping and reducing agents, respectively, to enhance the oxidative stability of CuNPs. The GA-capped CuNPs had a similar polygonal prismatic shape to uncapped CuNPs, with particle sizes ranging from 19.6 to 35.06 nm. Furthermore, the capped nanostructure demonstrated potent anti-*Salmonella typhimurium* activity, with a 27 mm zone of inhibition. Additionally, the GA-capped NPs efficiently reduced 95% of crystal violet and methylene blue within 30 min [39].

In another study, GA and chitosan were combined to coat liposomes nanoparticles loaded with 5I-1-indole, allowing them to serve dual functions as both an antifungal agent and a dye adsorbent for wastewater. At 25 μg/mL MIC, the coated liposome demonstrated potent antifungal action against the plant pathogen *Botrytis cinerea*. In addition, the adsorbent NPs successfully removed about 71.23% of the Congo red dye from water [40].

The influenza virus is a common respiratory pathogen that evades the immune system through slight changes in the hemagglutinin (HA) glycoprotein, a vital surface antigen targeted by most vaccines. In this line, GA polymer is used as a reducing agent of potassium permanganate in the biosynthesis of manganese dioxide nanoparticles (MnO_2_-NPs). The resulting nanosheets of MnO_2_-NPs were extremely thin with lateral dimensions, each measuring less than 100 nm. In addition, GA-MnO_2_-NPs showed significant antiviral efficacy against the influenza virus H1N1 in MDCK cells, with a 50% cytotoxic concentration (CC50) of 379 µg/mL and a non-cytotoxic concentration (NCTC) of 95 µg/mL. Notably, pre-infection therapy with the biosynthesized nanoparticles significantly alleviated the cytopathic effects induced by H1N1, achieving a 3.5 log decrease in hemagglutination and providing 69.7% cellular protection. Furthermore, molecular docking revealed that the high affinity of the GA polymer for the HA protein, as opposed to MnO_2_, exhibited no interaction with the targeted protein [32].

Carbon dots (C-dots) have arisen as a novel innovation in medicine and theranostics due to their superior biocompatibility, typical optical properties, good water solubility, and ease of surface functionalization, in contrast to semiconductor quantum dots such as CdTe and CdSe [41,42]. In recent years, chemical procedures for fabricating C-dots have been developed. Among the most well-known examples are microwave-mediated synthesis, graphite laser ablation, the thermal cracking of organic molecules, and graphite electrooxidation [43,44]. Furthermore, there are few studies on producing C-dots using natural plant materials as a carbon source. C-dot was synthesized from orange juice, jaggery, bread, and sugar [45,46]. Because these C-dots are created from natural materials, they are incredibly biocompatible and cost-effective for large-scale production. Thakur et al. used the microwave-assisted pyrolysis of GA to produce C-dots, which were subsequently conjugated to ciprofloxacin, a potent antibiotic. C-dots have a more than 90% loading capacity, making them an attractive vehicle for transporting large amounts of therapeutic payloads. The conjugation demonstrated synergistic antibacterial action against both Gram-negative bacteria and Gram-positive bacteria, associated with the sustained release of the loaded antibiotic [47].

#### 3.1.2. Cancer Therapy

As a photothermal therapy for liver preneoplastic lesions nodules (PLNs), GA was conjugated to AuNPs using trimeric glycine phosphine as a reducing agent. The GA-AuNPs produced were spherical, with particle sizes of about 15 to 18 nm and a zeta potential of −35.5 mV. Upon radiation with laser 800 nm, GA-AuNPs induced phototoxic effects on HepG2 cells, reducing cell survival and suppressing the histone deacetylase activity through interference with sulfhydryl groups on the histone surface. In diethylnitrosamine-induced PLN mice, treatment with GA-AuNPs paired with laser or without laser activation stimulated the extrinsic apoptotic pathway in cancer cells and inhibited liver PNL formation by activating caspase-3 and death receptor DR5. This was in addition to reducing TNF-α levels as an inflammatory marker and the placental glutathione S-transferase detoxification enzyme for carcinogens [48].

The same research team used GA–gold nanoparticles for photothermal therapy targeting lung tumors. The findings revealed that in the absence of laser radiation, the synthesized NPs were non-toxic to A549 cells at concentrations up to 8.6 µg/mL. Conversely, laser exposure induced GA-AuNPs to generate heat energy, leading to cellular damage and the reduced survival of A549 cells. Treating lung-tumor-bearing mice with GA-AuNPs/laser resulted in cell death within lung tumor tissues, accompanied by decreased levels of the inflammatory marker TNF-a and the angiogenesis mediator VEGF. In addition to an increase in lipid peroxidation, measured by MDA levels, this induces cellular oxidative stress and initiates the intrinsic apoptotic cascade in cancer cells by triggering caspase3 and releasing cytochrome C [49].

Hypoxia plays a vital role in oral tongue squamous cell carcinoma (OTSCC) therapy and is related to poor survival outcomes. The hypoxia/HIF-1α system is regulated by the overexpression of hypoxia-regulating miRNAs. To address this concern, spherical GA-AuNPs were formulated with a particle size ranging from 75 to 80 nm. CAL-27 cells treated with 400 µg/mL of GA-stabilized gold NPs exhibited dose- and time-dependent cytotoxicity, with IC50 values of 0.392 and 0.247 mg/mL for 24 and 48 h, respectively. In addition, DNA staining analysis revealed that GA-AuNP therapy primarily triggered early and late apoptotic cell populations at rates of 27.2% and 30.7%, respectively. Furthermore, the developed NPs mitigated hypoxia in CAL-27 cells through the suppression of HIF-1α and its regulatory miRNAs (miR-210 and miR-21), as well as its target gene c-Myc, an anti-apoptotic protein, at a concentration of 74.2 ug/mL (equal to 30% IC50 value) [50].

In a novel approach, ferrite nanoparticles were produced and subsequently encapsulated within GA with a core–shell structure to prevent aggregation. Following that, Au ions were immobilized on the core Fe_3_O_4_ NPs, which were then green reduced by GA’s oxygenation function to generate AuNPs, yielding a Fe_3_O_4_ NPs@GA/Au nanocomposite. The nanocomposite particles were globular, with homogenous sizes ranging from 20 to 35 nm. The findings revealed that the Fe_3_O_4_ NPs@GA/Au nanocomposite showed anti-leukemia properties in a dose-dependent manner against leukemia cell lines using the MTT assay. The bio-composite showed IC50 values of 156, 157, 198, 243, and 248 µg/mL against J.RT3-T3.5, TALL-104, Human HL-60, 32D-FLT3-ITD, and MOLT-3 cell lines [51].

In another study, GA was employed as a coating material for the formulation of gallic acid nanoparticles (Ga-NPs) through freeze-drying. The developed GA-coated Ga-NPs were spherical, with a 33 to 250 nm diameter and −15.2 mV zeta potential. The resulting NPs outperformed the DPPH-scavenging capacity and exerted remarkable antioxidant effects on RAW 264.7 cells by inhibiting nitric oxide compared to free Ga. Furthermore, nano-encapsulated Ga demonstrated antihypertensive efficacy, as verified by the increasing angiotensin-converting enzyme inhibition levels in vitro. Ga release from NPs was higher in an acidic medium than in alkaline buffers, making them effective against cancer cells. Furthermore, the generated NPs displayed antineoplastic action, with HepG2 and MCF7 displaying higher sensitivity compared to HT29 and MDA-MB231 cells, with an IC50 value half that of free Ga. This improved sensitivity could be attributed to the increased receptor selectivity facilitated by the chemical composition of GA’s galactose units, thereby promoting receptor-mediated endocytosis. This finding was supported by the observed increased selective uptake of Ga-NPs in HepG2 and MCF7 cells and their reduced metastasis potential [52]. GA was applied as a stabilizer in the eco-friendly production of nickel oxide nanoparticles (NiO-NPs) using the sol–gel approach. The bio-synthesized NPs were spherical, with a diameter of 59 nm. NiO-NPs displayed photocatalytic properties under UVA radiation. GA-stabilized NPs showed the 50% growth inhibition of glioblastoma cancer cell lines (U87MG) at a concentration of 10 µg/mL, as determined by the MTT assay [53].

Carbon dots were fabricated from GA using microwave-assisted heating and then purified with Sucrose Density Gradient Centrifugation. Afterward, purified C-dots were added to the primary seed solution to produce a spherical gold nanoparticle (GNPs)–C-dots complex to deliver doxorubicin. Doxorubicin was covalently and non-covalently linked to the complex with a high loading capacity due to the porous nature of the C-dots. An in vitro assay on the MCF-7 cell line proved that the prepared nanoplatforms could be used as a promising photothermal therapy and as cellular imaging agents [54].

#### 3.1.3. Drug Delivery

To improve the physicochemical characteristics of curcumin (Cur) in salt-containing food systems, Cur was loaded into nanoparticles utilizing GA as the wall material. The highest Cur loading capacity in GANPs was 0.51 μg/mg, with particle sizes ranging from 457.4 to 470.1 nm. Remarkably, the findings revealed that even at 0.5 M sodium chloride, around 80% of the Cur amount in the NPs was retained. Furthermore, Cur-loaded GANPs were resistant to UV radiation, with 82% of the Cur remaining encapsulating within NPs after 5 h of light exposure. This UV resistance was ascribed to the GA protein moiety, which absorbed incident light. Notably, the release of Cur from GANPs occurred during intestinal digestion, and the liberation pattern was more responsive to changes in pH than the presence of protease enzyme [33].

In recent work, Cur was encapsulated into nanoparticles formulated from GA and sodium alginate using ionotropic gelation. The encapsulated NPs were spherical, with a particle size ranging from 10 to 190 nm and a zeta potential of −15 mV. Notably, GA/SA NPs achieved a high Cur entrapment efficiency, reaching 89% due to GA’s emulsifying and gelling features. The Cur-loaded NPs exhibited potent antioxidant action in a dose-dependent manner compared to free Cur. Furthermore, the Cur-loaded GA/SA NPs displayed comparable antiproliferative activity against HT29, MCF7, and A549 cell lines when compared to free Cur. However, Cur/GA/SA NPs exerted higher toxicity against HepG2 cells than free Cur owing to the selective binding of the GA galactose units to the ASGPR receptors on human hepatocytes [55].

Luteolin was encapsulated into a nanocomposite composed of *Stauntonia brachyanthera* seed albumin (SBSA) as the primary material and stabilized with GA and carboxymethylcellulose (CMC) using a health-induced self-assembly approach. The smallest particle size of the spherical nanocomposite, measuring 204 nm, was attained when the mass ratio of SBSA to GA was 2:1. Furthermore, the zeta potential of SBSA/GA/CMC NPs dropped to −30 mV at a mass ratio of 15:1 for SBSA to CMC. Interestingly, adding CMC into the colloidal system did not affect its storage stability since both colloidal systems (SBSA/GA and SBSA/GA/CMC) remained stable for one month without precipitation. This nano delivery system demonstrated a good luteolin EE of 84.0%, leading to increased luteolin bioavailability up to 46.8%, and demonstrated stability under varying pH and salt conditions [56].

To improve the bioavailability of epigallocatechin (EGC), it was loaded into zein NPs using an antisolvent precipitation technique, and then the loaded NPs were gradually added to GA solutions to produce a zein–GA–EGC nanocomposite. The produced zein–GA–EGC nanocomposite displayed a particle size of 128.0 nm, with a 75.2% EE for EGC. Under simulated gastric fluid, EGC displayed delayed liberation from the nanocomposite, with only 16.4% released after 90 min, compared to the 25.5% released from zein NPs. However, in simulated intestinal fluid, EGC was released rapidly in the first 60 min; then, it continued in a sustained pattern [57]. Similar outcomes were reported when using a zein–GA nanocomposite to encapsulate a rutin-rich extract, indicating the potential use of this approach for the controlled release of bioactive substances in the gastrointestinal system [58].

Beta (β-lactoglobulin; Lg), a small-molecular-weight protein, produced a compact structure with GA as a protective carrier for EGC. At an EGC concentration of 0.2 mg/mL, the EE of the β-Lg/GA nanocomplex was 84.5%, ascribed to GA incorporation, which improved the interaction between polymers and EGC within the complex. In vitro studies showed that a β-Lg/GA nanocomplex demonstrated significant antioxidant activity by increasing the proton released from EGC via hydrogen bonds formed between β-Lg, GA, and EGC. Furthermore, the generated nanocomplex displayed good storage stability, photostability, and gastric stability, with only 21% being released after 60 min in SGF, against 86% released after 3 h in SIF [59].

Quercetin (Qr), at concentration of 60 μM, was encapsulated into a nanoformulation made of chitosan and GA. This nanostructure was prepared using an ionic gelation process, and had a diameter ranging from 267.3 to 493.2 nm and an EE of over 90%. In vitro studies showed that the strong electrostatic interaction between cationic Qr/CS/GA-NPs and the anionic mucin layer resulted in superior mucoadhesive properties, further enhanced with an elevated GA concentration. The results revealed that the improved cellular adhesion was associated with greater permeation across epithelial cells via simple diffusion, thereby boosting the antioxidant properties of absorbed Qr in an intestinal Caco-2 cell model and in rat blood [60].

To improve luteolin bioavailability, zein–GA–tea polyphenol NPs (ZGT) were produced using an anti-solvent precipitation assay as a delivery system. The produced nanoparticles were smooth and spherical, with a particle size of 202 nm at a zein to GA mass ratio of 1:2. Incorporating tea polyphenols at a concentration of ≤0.2 mg as a cross-linking stabilizer, together with 10 mg of luteolin, resulted in 85.0% encapsulation. Furthermore, the ZGT nanoplatform enhanced luteolin retention after 4 weeks of storage to approximately 91.2%, compared to 84.3% in the nano platform without tea polyphenols. In addition, the colloidal NPs demonstrated excellent antioxidant efficiency, with the sustained release in SGF being 26.4% compared to 60.7% in SIF [61]. The influence of adding calcium ions to the ZGT nanoplatform was also explored. The results revealed that adding low concentrations of calcium ions (2–4 mmol) as a cross-linking stabilizer promoted favorable physiochemical characteristics under various conditions such as temperature, pH, ionic strength, and storage. Moreover, it improved gastric stability and the antioxidant activities of NPs [62].

An ionic gelation approach was used to generate a nanocarrier between chitosan (CS) and GA as a delivery system for saffron extract. The resulting nanocomplexes were spherically formed, with smooth surfaces and particle sizes ranging from 183 to 295 nm. Moreover, the entrapment efficiency increased to 52.3% with higher concentrations of CS and GA within the nanocarrier. Nevertheless, an increase in the amount of saffron extracted led to a decrease in EE. During the first hour, saffron liberation from the nanocomplex increased exponentially to 80% under an acidic pH. This release pattern was attributed to the loss of nanocarrier structure integrity caused by the increased CS solubility and the high porosity resulting from GA chain disarrangement. At a neutral pH, around 70% of saffron was released from the nanocarrier after 4 h [63].

To mitigate the oxidative damage caused to intestinal cells, water-soluble biopolymers such as Gum Arabic, alginate, xanthan, and tragacanth were coupled with soy phospholipid and whey to formulate an antioxidant delivery system for gingerol. The generated vesicles were primarily unilamellar, with small particle sizes around 100 nm, a high negative surface charge, and a 94% encapsulation efficiency. The findings demonstrated that the composite biopolymers and whey liposomes exhibited great gastrointestinal stability and were non-toxic, particularly in the case of alginate and GA–whey vesicles, where the cell survival was almost 100% even at the highest formulation concentration. In addition, tragacanth–whey liposomes were the most effective in protecting intestinal cells from the oxidative damage induced by hydrogen peroxide. Interestingly, combining GA and tragacanth gum with whey liposomes resulted in fast wound healing and the full closure of wounds after 72 h of treatment [64].

During the fabrication of liposomes from soy phosphatidylcholine using the ethanol injection technique, various concentrations (0–4%) of sodium alginate (SA) and GA were added to the organic phase to evaluate their impacts on the curcumin (Cur) loading capacity. The results showed that a higher interaction between GA and the liposome surface resulted in smaller particle sizes of 148 nm and 0.15 PDI achieved at a 2% GA concentration, leading to high Cur encapsulation. Nevertheless, low GA concentrations disrupted the liposomal internal lamellar structure, resulting in poor Cur retention (~20%) and limited storage stability at 4 °C. Even though SA liposomes had a large particle size of 299 nm and a high PDI of 0.6, SA liposomes were preferable for long-term storage and retained over 70% of their Cur content after 7 days of storage. This may be attributed to the high viscosity of SA, which inhibits particle aggregation, flocculation, and mobility, thus ensuring the integrity and stability of the liposomal structure over time [65].

#### 3.1.4. Bioimaging

For CT imaging, gold NPs were produced utilizing a pulsed laser ablation process, with GA serving as both a stabilizing agent and contrast enhancer. GA-AuNPs displayed a smaller size, ranging from 1.85 to 0.99 nm, and were spherical in shape. The results indicated that adding 20 mg of GA induced a blue shift in the plasmon peaks, resulting in a shorter wavelength of 514 nm, as opposed to the red shift observed at 521 nm for pure AuNPs. A blue shift towards lower wavelengths was observed under high energy conditions exceeding 200 mJ (milli Joules). In addition, the number of laser pulses had a minimal impact on the particle size, which remained around 3 nm. In addition, increasing the concentration of GA-AuNPs to 54 ppm improved the brightness, CT number, and overall image quality [66].

#### 3.1.5. Biosensing and Enzyme Immobilization

Ag NPs stabilized with GA were bio-synthesized as a chemical sensor for hydrogen peroxide and glucose detection. The produced NPs were monodispersed and ranged in size from 15 to 20 nm. The developed sensor demonstrated high sensitivity in detecting hydrogen peroxide in water and glucose in human blood, with limits of detection of 11.7 nM and 0.13 µM, respectively. GA-Ag NPs coated on filter paper exhibited remarkable SERS activity for H_2_O_2_ detection, achieving a detection limit of 0.56 µM [67].

Magnetite (Fe_3_O_4_) NPs coated with GA were employed as a magnetic support for the immobilization of trypsin enzyme. Initially, the Fe_3_O_4_ NPs were coated with GA, followed by the activation of the OH^−^ groups on the GA-coated Fe_3_O_4_ NPs using glutaraldehyde. This activation facilitated the reaction among the trypsin enzyme’s OH^−^ and amino groups, enabling their immobilization via strong covalent interaction. Remarkably, the immobilization procedure resulted in the enhancement of enzymatic activity over a broad pH range (4–11), across temperatures ranging from 20 to 80 °C, and improved the solvent stability compared to the free enzyme. Furthermore, reusability tests showed that the immobilized enzyme restored over 93% of its activity for up to 15 cycles (Table 4) [68].

## 4. Gum Arabic-Based Hydrogels

**Figure 10:**
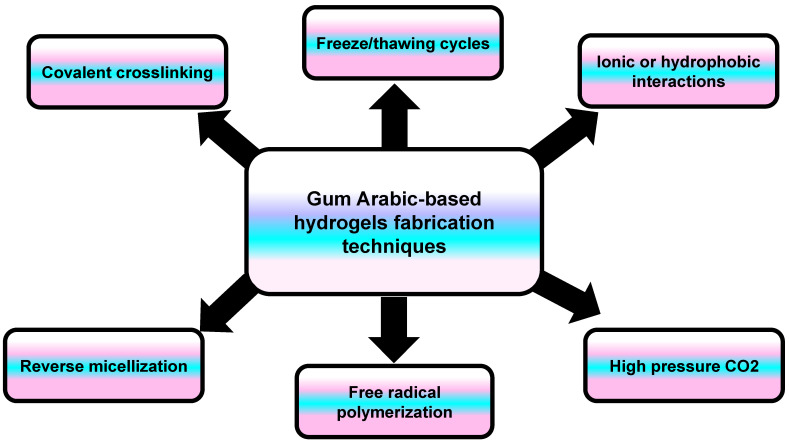
An illustration showing the different techniques involved in the synthesis of Gum Arabic-based hydrogels.

A hydrogel is a three-dimensional network structure composed of polymeric monomers that are highly capable of absorbing water. The hydrogel’s structure contains either the same polymer (homopolymer) or a different one (copolymer), which are cross-linked to prevent their dissolution when exposed to water [69]. Hydrogels are developed through either chemical cross-linking (chemicals, grafting), physical cross-linking (H-bonding, complex coacervation), or radiation cross-linking (radiation in paste, aqueous state radiation) [70]. In general, the hydrogel scaffold properties depend on the fabrication material (composition/concentration) and the cross-linking method (Figure 10) [71]. Hydrogels mimic natural living tissue owing to their high water content and softness. Due to this similarity, they can be employed in many biomedical applications, such as tissue engineering, wound dressing materials, drug delivery, and molecular imprinting [72].

### 4.1. Applications of GA-Based Hydrogels

#### 4.1.1. Tissue Regeneration

Hydrogels are ideal scaffolds for tissue engineering due to their extremely porous interconnected structure, mechanical strength, swelling, and cell adhesion capabilities [73]. Naturally produced biomaterials have recently received great interest in material science due to their resemblance to ECM, their innate biocompatibility, and the limited probability of immunological reaction and immune rejection occurring compared to human-made materials [74]. GA was first employed as a surfactant to improve the porosity and swelling of chitosan hydrogel, enhancing the penetration and viability of seeded fibroblastic cells in tissue engineering applications [75]. In a different approach, modified GA was used as a non-toxic cross-linker for a gelatin-based hydrogel. GA was first oxidized with periodate to form GA aldehyde (GAA), and then the hydrogel was produced by a Schiff’s base reaction between the aldehyde groups of GAA and the free amino groups of gelatin. The prepared scaffold showed a high swelling ratio (66%), while the degradation study showed stability over four weeks. The prepared scaffold represented a suitable matrix for spheroid cell culture application [76]. A GA microgel was prepared using the reverse micellization technique with slight modification. The microgel was formed when the hydroxyl group on the inter and intra chains of different GA monosaccharide units found in the reverse micelles core bound with divinyl sulfone (cross-linking agent). Furthermore, the hydrogel gained a significant antibacterial property when diethylenetriamine and taurine (TA) were added as chemical modifiers. The microgel was found to be hemocompatible, biocompatible, and highly stable at physiological pH, suggesting that it could be used in the biomedical field [77,78]. The same technique was used to prepare a GA hydrogel/metal sulfide composite. This microgel was used as a template for in situ ZnS or CdS NP preparation based on the interaction between metal ions and the hydroxyl and carboxylic acid residues found in GA chains. The nanocomposite displayed a remarkable antimicrobial and antioxidant capacity, indicating a high potential for tissue engineering, wound healing, and drug delivery applications [79].

Makar et al. developed a novel hydrogel composed of GA, chitosan, and nano-hydroxyapatite through physical and covalent cross-linking with FeCl_3_ and genipin, respectively. The genipin cross-linked hydrogel had more interconnected pores than the FeCl_3_-cross-linked one, which enhanced cellular adhesion and ingrowth. It also had higher compressive strength, an important criterion for hard bone regeneration. Furthermore, using the genipin cross-linker increased the hydrophilicity of the hydrogel, as evidenced by the increased water absorption necessary for nutrient and metabolite diffusion. The bone regenerative potential of the two prepared hydrogels was confirmed in vivo using a rat critical-size calvarial defect model, where both hydrogels displayed new bone regeneration [80].

#### 4.1.2. Drug Delivery

A natural hydrogel is a 3D polymeric network that is biodegradable and biocompatible. It can be endowed with stimulus responsiveness to environmental conditions such as pH and temperature [81,82]. Furthermore, the degree of cross-linking in the hydrogel matrix can modify its porous structure, affecting the drug release kinetics [83,84]. These distinctive physical properties of hydrogels have led to extensive interest in their use in drug delivery applications [85]. From this perspective, periodate oxidized GA was used as a green cross-linker to form a smart PVA-based hydrogel that was responsive to pH change loaded with folic acid. The prepared hydrogel displayed a sustained release profile with a higher release percentage at an alkaline pH (7.4) than at an acidic pH (2.1) due to the collapsed hydrogel form caused by high acidity. The hydrogel showed a UV-photoprotective effect on the loaded folic acid, making these hydrogels a suitable candidate for the sustained delivery of folic acid [84]. In a similar approach, a nano curcumin injectable hydrogel was developed using succinic anhydride-modified chitosan cross-linked with a multialdehyde GA. This hydrogel showed pH-responsive drug release at a tumoral acidic pH compared to at physiological conditions. As a result, the prepared biodegradable injectable hydrogel is a promising delivery system for anticancer drugs treating locally accessible cancers [86]. Ibuprofen-loaded chitosan-PC90G with a GA hydrogel was prepared using a green synthesis method that involved ionic interaction and freeze–thawing cycles of PVA/chitosan. This nanoconjugate hydrogel reduced the drug crystallinity and prolonged the release profile, which allowed effective transdermal drug delivery [87]. In another study, thyme essential oil loaded into nanoliposomes was infused into a natural hydrogel composed of pea protein and GA. The whole composite demonstrated good water retention, swelling and mechanical strength, making it a suitable matrix for the controlled oral delivery of essential oil as it can protect the oil from degradation in simulated gastrointestinal conditions [88].

In another study, GA and tragacanth gum (TG) were utilized as reducing agents to prepare Ag NPs. The GA-TG-Ag NPs were then grafted into an acrylamide polymer matrix to generate a composite hydrogel for the sustained oral drug delivery of the antibiotic meropenem. The composite displayed good thermal stability due to the presence of Ag NPs and the grafting process. The controlled and slow release of the drug was observed owing to the drug/polymer interaction, network density, and hydrogel porosity. Furthermore, the composite demonstrated antibacterial, antioxidant, and mucoadhesive properties, representing a promising candidate for colon infection drug delivery [89]. A GA hydrogel cross-linked with epichlorohydrin, reinforced with microcrystalline cellulose, and loaded with sulfadiazine was prepared for antimicrobial applications. The cross-linking was carried out via the nucleophilic interaction of the alcoholate anion, and the hydroxyl groups of both polysaccharides were covalently bonded to create a new epoxide via chloride displacement. The generated hydrogel showed regulated drug release for up to 8 h with pH responsiveness, indicating a promising platform for sulfadiazine administration [90].

A co-assembled dipeptide hydrogel was fabricated from an alkyl-chain-modified dipeptide [C13-tryptophan-tyrosine (C13-WY)] with GA to improve the mechanical stability of the peptidyl hydrogel. The fabricated hydrogel formed more β-sheet structure and antiparallel hydrogen bonds than the self-assembled peptidyl hydrogel. The co-assembled C_13_-WY-GA displayed minimal cytotoxicity to ovarian carcinoma (A2780) and HeLa cells. Therefore, it was utilized to encapsulate the hydrophobic drug (docetaxel), which demonstrated sustained drug release [91]. An injectable hydrogel was developed by Schiff base bonding between the polyaldehyde GA and carboxy methyl chitosan. Then, doxorubicin-loaded graphene oxide NPs were entrapped within the gel matrix to enhance its mechanical properties. The hydrogel exhibited self-healing properties and pH-responsive degradation and drug release. The biodistribution test revealed that this hydrogel has a good retention time and slow drug release at the tumor site. As a result, the developed injectable hydrogel could serve as a promising delivery vehicle for cancer therapy [92].

#### 4.1.3. Wound Dressings

Hydrogels have a structure comparable to the natural extracellular matrix, which not only mimics the milieu of human tissues but also serves as a moist environment for wound healing [93]. Furthermore, hydrogel dressings allow for gas exchange while preventing infections by forming a physical barrier against microbial invasion, making them even more ideal for wound dressing applications [94]. In this regard, a novel polyacrylic acid-co-Al^3+^-co-modified GA self-healing hydrogel was fabricated by the free radical polymerization of acrylic acid and 1-vinyl-3-butylimidazolium. Subsequently, GA was modified with octane succinic acid, which introduced carboxyl groups and enabled dynamic physical cross-linking via ionic interaction with Al^3+^, as well as hydrogen bonding with acrylic acid. The prepared hydrogel showed good mechanical properties and antibacterial activity against *S. aureus*, *E. coli*, and *C. albicans*. In addition, it displayed accelerated healing properties, providing a promising dressing material for wound healing [95].

Pectin and GA were cross-linked using Ca^2+^ ions, leading to the GA/pectin hydrogel loaded with naringin, a natural flavanone glycoside, being formed with an egg-box-like structure. This physical cross-linking was facilitated by ionic interaction between the glucuronic acid (COO^−^) found on the surface of the used polysaccharides and the cationic divalent calcium ions. Pectin/GA hydrogel was utilized to encapsulate naringin, which possesses anti-inflammatory, antioxidant, and anti-apoptotic effects. Afterward, the hydrogel was evaluated for its wound-healing ability in an in vivo rat model. The results showed enhanced angiogenesis, reepithelization, and collagen deposition. In addition, the hydrogel showed potent antioxidant and anti-inflammatory actions, which demonstrated the superior potentials of the prepared hydrogel in wound dressing applications [96].

Another hydrogel was fabricated based on the development of interpenetrating polymeric networks of collagen/GA matrix cross-linked with polyurethane. The addition of GA enabled the development of a polymeric network with a faster gelation time through the formation of intermolecular hydrogen bonds. In addition, GA afforded an amorphous surface semi-interpenetrating hydrogel with improved matrix reticulation, swelling, mechanical strength, and thermal degradation. Additionally, GA incorporation prolonged the release of a model drug (ketorolac), improved the hemocompatibility, and enhanced the antibacterial effect. The hydrogel matrix demonstrated no cytotoxicity and reduced the secretion of inflammatory markers (α-TNF), indicating its potential for wound dressing [97].

Keykhaee et al. developed a multifunctional alginate-GA hydrogel cross-linked with Ca^2+^ ions, in which nerve growth factor-loaded mesoporous silica NPs and carnosine were incorporated to improve the hydrogel’s therapeutic properties. While incorporating silica NPs improved the hydrogel’s mechanical and viscoelastic properties, carnosine addition (<5 mg/mL) increased the number of pores in the hydrogel structure and subsequently decreased its mechanical strength. An in vivo study on a full-thickness wound in streptozotocin-induced diabetic rats showed that the fabricated composite significantly reduced inflammation and induced angiogenesis, promoting wound healing [98]. Similarly, an alginate-GA hydrogel was prepared using the conventional external gelation mechanism, with CaCl_2_ as a source of Ca^2+^, to immobilize S-acetamido methyl Cys20, 31-EGF peptide. This peptide mimics growth factor signaling to modulate cellular activities such as proliferation, differentiation, migration, and tissue regeneration. The immobilized peptide does not affect the hydrogel’s physicochemical properties, while the hydrogel regulates the peptidyl release sustainably. In an in vitro examination of its wound-healing capacity, the prepared hydrogel indicated enhanced fibroblast migration and proliferation, reduced inflammation, and increased angiogenesis [99]. 

Using coordination chemistry, a multifunctional adhesive hydrogel was prepared using a Fe^3+^-mediated chemical combining GA and gelatin for matrix preparation. The hydrogel was loaded with allantoin, an FDA-approved product with wound-healing abilities. The hydrogel demonstrated numerous appealing features, including fast gelation, high porosity, a high water content, adhesiveness, and injection potential. More importantly, an in vivo study indicated that the wound contraction percentage was 83% after 14 days, with no leakage, infections, or inflammation [100].

#### 4.1.4. Biomedical Devices

Hydrogel flexibility and stretchability are valuable mechanical properties in case of exposure to a repetitive strain condition. Furthermore, hydrogel biocompatibility and similarity to native soft tissue are essential requirements for potential application in the biomedical field [101]. Conductive materials can be used in biomedical devices by integrating them within a self-healing hydrogel, creating a conductive hydrogel for sensing devices [102]. GA was chemically modified with glycidyl methacrylate (GMA), then cross-linked by methylene bisacrylamide via a free radical reaction. Chitin nanowhiskers (CtNWs) were then added to the hydrogel to increase its rigidity and decrease the swelling capacity. However, there was no linear correlation between these properties and the amount of CtNWs. Hydrogels reinforced with CtNWs are suggested to have great application prospects in biomaterials and biomedical devices [103].

Tomić et al. developed a PVA hydrogel with enhanced conductivity by adding different ionic polysaccharides, including GA, xanthan, and sodium carboxymethyl cellulose. The electrical conductivity was further boosted by adding graphene-based fillers (Reduced graphene oxide, graphene nanoplatelets, and activated carbon black), which participate in reversible dynamic bonding. The obtained composite represents a promising polysaccharide-based hydrogel for wearable devices for healthcare applications (Table 5) [104].

## 5. Gum Arabic-Based Nanofibers

Nanofibers (NFs) are fibers with diameters in the nanometer scale, typically from 1 nm to 100 nm. They have unique characteristics due to their high surface area-to-mass ratio, superior mechanical performance, tunable size and morphology, and flexibility in surface functionalities [105]. Additionally, NFs form an excellent porous mesh that creates an ideal three-dimensional (3D) network environment, making them highly suitable for biomedical applications [106]. NFs can be utilized in wound dressing [107], biosensing [108], controlled drug delivery [109], cancer therapy [110], antimicrobial application [111], tissue regeneration [112], and cardiovascular disease treatment strategies [113]. NFs can be produced from various materials using electrospinning, phase separation, and self-assembly [114].

Among the various methods used to fabricate NFs, the electrospinning technique excels regarding the production of homogeneous and versatile NFs from natural and synthetic polymers, which are suitable for biological systems applications [115]. Two significant advantages of the electrospinning technique are its cost-effectiveness and high drug-loading capacity, making it the most utilized technique for the fabrication of NFs [116]. The morphology and quality of the developed electrospun NFs can be controlled by different factors such as the flow rate of the solution, the polymer concentration, solvent volatility, surface tension, and the applied voltage [117]. NFs can be fabricated from synthetic polymers such as polyglycolic acid (PGA), polyvinyl alcohol (PVA), polylactic acid (PLA), poly (ethylene oxide) (PEO), and poly (e-caprolactone) (PCL). They usually possess good mechanical strength and flexibility. Although NFs fabricated from synthetic polymers are biocompatible, they lack the bioactivity displayed by natural polymers [118]. Natural polymers are more biocompatible and have good cell surface affinity, in addition to low immunogenicity compared to synthetic polymers [115]. Moreover, natural polymers mimic the extracellular matrix (ECM); thus, they represent superior and effective platforms for biological processes and cellular interactions [119]. Therefore, blending natural polymers with synthetic polymers is a favorable strategy to enhance the synthetic polymer’s biocompatibility property. Combining these polymers merges their characteristics features, resulting in an optimal performance for intended applications [118,120]. GA has been successfully blended with synthetic polymers to produce electrospun NFs [121]. GA is a water-soluble polymer; thus, it is effectively used for drug delivery and biomedical applications. GA can be utilized to fabricate scaffolds such as NFs for diverse applications in the biomedical and food technology sectors [122].

### 5.1. Applications of GA-Based NFs

#### 5.1.1. Tissue Regeneration

Electrospun PVA/GA NFs were synthesized to produce a biomimetic extracellular matrix (ECM) that can improve in vitro cell growth and promote precision medicine [123]. The nanocomposite mat was analyzed and characterized by SEM, FTIR, and confocal laser scanning microscopy (CLSM). The results revealed a uniform and smooth surface with an average diameter of about 227 nm. The obtained PVA/GA NFs demonstrated good biocompatibility, as the NFs exhibited no change in their cellular morphology upon their in vitro incubation with cells, confirming their potential as a biocompatible scaffold. Afterward, the same scaffold was further functionalized with GA-coated Au NPs to examine its anticancer efficacy against B16-F10 (murine metastatic melanoma) cells. The results showed improved cellular inhibition [123]. However, more detailed studies are required both in vitro and in vivo to study the exact mechanism of cellular proliferation inhibition.

Blended 3D NFs comprising zein, Calendula officinalis (*C. officinalis*) extract, GA, and poly (ε-caprolactone) (PCL) were synthesized by electrospinning for skin tissue engineering purposes [124]. Morphological examination of the scaffold revealed a cone-like structure with interconnected pores and a 3D structure as a result of the high relative humidity and repulsions between adjacent fibers [124]. In addition, these 3D scaffolds exhibited good degradability and strength to an extent, allowing improved cell growth, proliferation, adhesion, and penetration. Moreover, these NFs demonstrated significant hydrophilicity and high porosity (approximately 94% porosity), with an average pore size greater than 9 μm [124]. In vitro studies demonstrated that the PCL/Zein/GA/C officinalis 3D scaffold with nanofibrous/net morphology supported excellent cell growth and attachment, suggesting that these 3D fabricated scaffolds made from PCL, zein, GA, and C. officinalis are promising candidates for repairing skin defects.

#### 5.1.2. Wound Healing

Cheng C. et al. prepared keratin (K), GA, and γ-polyglutamic acid (PGA) NFs loaded with Cur via the blending electrostatic spinning approach [125]. The fabricated NFs were precisely characterized by scanning electron microscopy (SEM), fluorescence microscopy, differential scanning calorimetry (DSC), Fourier transform infrared (FTIR) spectroscopy, and X-ray diffraction (XRD). Additionally, the antimicrobial effect against Staphylococcus aureus and the wound-healing mechanism of action of KGP-C NFs were evaluated. The results showed that Cur was successfully encapsulated within the NF matrix, with a uniform diameter, satisfied water absorption rate, and enhanced mechanical properties. Furthermore, Cur-loaded NFs effectively inhibited *S. aureus*, achieving an impressive inhibitory rate of up to 70.3%, associated with excellent biocompatibility. The topical application of KGP-C NFs significantly enhanced wound healing by boosting the expression of TGF-β1 and VEGF while downregulating the pro-inflammatory factor IL-6, causing a subsequent increase in the re-epithelialization rate and improved wound contraction [125].

Mats comprising GA/PVA NFs were fabricated by electrospinning and coated with polycaprolactone (PCL); then, they were loaded with Ag NPs [126]. The Ag NPs showed good stability due to the intramolecular and intermolecular forces afforded by GA, as it formed a hydrogen bond network within the polymeric chains. The force formed between the network and the polymer with AgNPs originated from the carboxylate group (-COOH) of the polymeric chains [127]. The PCL-coated NF mat demonstrated excellent water stability and a high water absorption capacity. Additionally, the mat’s structure illustrated an optimal porosity and appropriate water vapor permeability, rendering it a promising candidate for wound dressing applications. The antimicrobial activity of the AgNPs@GA-PVA-PCL mat against *Escherichia coli*, *Staphylococcus aureus*, *Pseudomonas aeruginosa* and *Candida albicans* showed a distinct zone of inhibition in and around the AgNP-loaded mat for all tested microbes, measuring 2.5 mm for *S. aureus*, 2.9 mm for *E. coli*, 6.3 mm for *P. aeruginosa*, and 2.6 mm for *C. albicans* [126]. As *P. aeruginosa* and *S. aureus* are major wound-infecting pathogens [128,129], the usage of these electrospun mats would be highly suitable for wound-dressing applications. The antimicrobial mechanisms could be due to the interaction of AgNPs with the cell wall and membrane, with the Ag NFs then penetrating the cell and causing membrane damage and the leakage of the cellular contents. In addition, the production of reactive oxygen species (ROS) and free radicals by AgNPs can induce an apoptosis-like response, destroy cell membranes, and cause DNA damage. Moreover, the binding of AgNPs to sulfur and phosphorus groups in DNA results in DNA damage and the disruption of DNA transcription and translation [130,131]. Additionally, the MTT assay was assessed to evaluate the cytotoxicity of the mat and its biocompatibility on the mouse embryonic fibroblast cells, and results showed that these NFs mats had excellent biocompatibility and an elevated proliferation of fibroblast cells [126].

#### 5.1.3. Antibacterial and Anti-Biofilm Activities

A blend of the natural polymers GA and pullulan (GA/Pul) was utilized to fabricate biocompatible nanofibrous mats [132]. These natural polymers have superior advantages over synthetic ones as natural polymers are hydrated polysaccharides that can protect the skin from dryness. In contrast, synthetic polymers have deleterious effects on the skin, which might cause skin dryness and irritation [133]. Blended natural fibers of GA/Pullulan (GA:Pul) were loaded with ZnO NPs using centrifugal spinning, and then the efficacy of the prepared biopolymeric ZNO@GA/Pul NFs was examined against *Acne vulgaris* [132]. *Acne vulgaris* is a major skin condition affecting a large portion of the population, around 85% of young individuals, while teenagers are the main group affected by this condition [134]. Topical application is widely used for mild and moderate cases, requiring high doses to be effective, which might cause burning, itching, and erythema of the skin [135]. Fabricated NPs were characterized by electron microscopy energy-dispersive X-ray fluorescence spectrometry to examine the NFs’ composite homogeneity. Subsequently, the antibacterial properties of the fabricated NFs were evaluated against *Cutibacterium acnes* and *Staphyloccocus epidermidis*. Antibacterial examination showed that *S. epidermidis* was significantly affected by ZnO NPs at concentrations above 1.5 wt. %, while *C. acnes* inhibited bacterial growth at a lower concentration of 0.6 wt. % ZnO NPs, highlighting their remarkable antibacterial properties [132]. This antibacterial activity is mainly due to the potential of ZnO NPs to induce the production of reactive oxygen species (ROS) after their internalization and Zn^2+^ release. Consequently, ROS led to a detrimental impact on bacterial DNA, causing their death [136]. Another factor contributing to the inhibition of bacterial growth is the shape and size of the ZnO NPs. The particles were mostly spherical, with small diameters, allowing them to penetrate bacterial membranes easily, thereby enhancing their antibacterial effectiveness [137]. Therefore, these blended NFs were considered an effective treatment approach for Acne vulgaris.

A tyrosol (TYS) functionalized chitosan–gold nanocomposite was incorporated into PVA/GA NFs via electrospinning to suppress *Candida* biofilm formation [138]. It was found that the addition of TYS to the growth medium led to inhibitory activity on both planktonic and sessile forms against single and mixed species: *Candida*, *Streptococcus mutans*, and *Pseudomonas* [139]. TYS-AuNP@ was incorporated into PVA/GA NFs to afford the direct delivery and sustained release of the loaded composites to the infected area and ensure long-lasting activity. The fabricated NFs were examined using a field emission scanning electron microscope (FESEM) and FTIR analyses to evaluate successful encapsulation. The results confirmed the successful encapsulation of TYS within the NFs, with sustained drug release and the long-time stability of TYS in the medium. Moreover, the prepared mats exhibited good fluid absorption capabilities, besides growth and biofilm inhibition against *Candida,* with about 60–70% biofilm disintegration with the use of 10 mg of TYS-AuNP@PVA/GA NFs. Additionally, the fabricated NFs significantly decreased the hydrophobicity index and ergosterol content compared to untreated cells. Consequently, nanocomposite-loaded NFs have been introduced as a potential material for controlling fungal colonization and promoting wound healing [138].

Geraniol loaded β-cyclodextrin was incorporated into PVA/GA NFs using an electrospinning technique [140]. β-Cyclodextrin was incorporated with geraniol to achieve efficient encapsulation, according to the method outlined by Aytac et al. [141]. Geraniol-based NFs showed a broad size distribution of about 142 ± 61 nm because of the non-uniform distribution of the inclusion complex of β-cyclodextrin and geraniol within the NFs. This non-uniformity might be due to the thermodynamic incompatibility between the inclusion complex of β-cyclodextrin and geraniol and the polymer solution, shown by surface tension effects [142]. The conductivity results indicated that the geraniol@ GA/PVA NF polymer solution had lower conductivity compared to the blank NF solution (GA/PVA). This reduced conductivity necessitates a higher driving force to produce the NFs. This is because the increased polymer chain entanglement results in the production of NFs with thicker diameters [143]. Moreover, the fabricated NFs caused more controlled drug release with improved hydrophilicity and mechanical properties. Not only did the GA/PVA matrix enhance the encapsulation of geraniol, but it also facilitated its sustained release. More importantly, fabricated NFs showed a potent suppressive effect against fungal biofilms via the effective inhibition of *Candida albicans* and the *Nakaseomyces glabrata* sessile form, and the eradication of about 85% of the biofilm at 10 mg/mL of NFs. Furthermore, the fluorescence microscopy examination illustrated the lethal effect of the NFs on *Candida albicans*. An in vitro cytotoxicity evaluation by MTT assay revealed no toxic effect on CHO epithelial cells [140]. Therefore, the engineered NFs can be extensively utilized in drug delivery applications, in addition to being used in antifungal infections as transdermal substitutes.

#### 5.1.4. Food Packaging

The incorporation of probiotics into edible films or fibers represents a promising innovation in active packaging for food preservation and health benefits. However, overcoming the challenges of maintaining probiotic viability in diverse environmental conditions remains crucial for the widespread application of these films [144]. In this regard, a recent study developed a NF film fabricated from Gum Arabic (GA), pullulan (PUL) and chia mucilage protection solution (CPS) to incorporate *Lactobacillus bulgaricus* (LB) via the blend electrospinning technique [145]. *Lactobacillus bulgaricus* (LB) is one of the active antibacterial ingredients, accounting for the increased food shelf life due to its outstanding antibacterial properties and its related health benefits. Furthermore, GA and PUL exhibited good emulsification and film-forming properties, whereas CPS displayed a comprehensive polysaccharide network structure and a remarkable amount of dietary fiber. Microscopic visualization revealed that the presence of CPS in the matrix of the NFs significantly increased the survival of LB, reaching a live count of about 7.62 log CFU/g after 28 days of storage at 4 °C, with an increase of nearly 1 log CFU/g in comparison to NFs without CPS. Additionally, the measurement of total volatile basic nitrogen (TVB-N), pH, and total viable count (TVC) demonstrated that the antibacterial biopackaging film prolonged beef’s shelf life by two days at 4 °C [145]. Further research is needed to thoroughly investigate the metabolic reactions of probiotics within fiber films to fully understand and enhance their beneficial properties for food preservation applications (Table 6).

## 6. Gum Arabic-Based Membranes or Scaffolds

**Figure 11:**
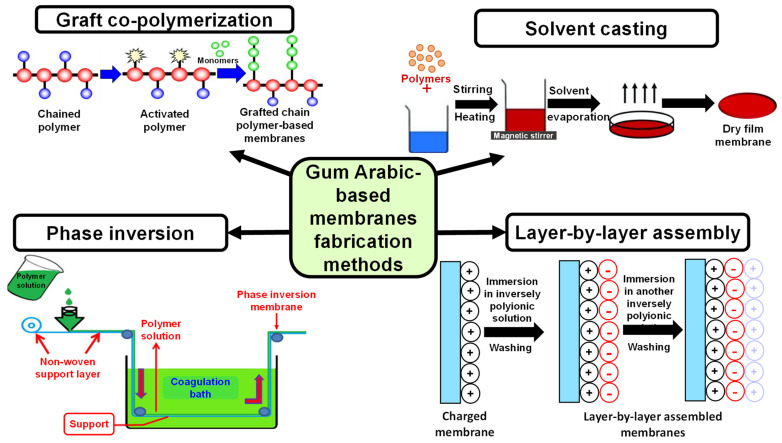
Different methods utilized in the synthesis of Gum Arabic-based membranes.

GA possesses diverse, outstanding features that enable it to be a unique polymer for scaffold fabrication, including antibacterial, expectorant, astringent, and immunosuppressive effects. In the pharmaceutical sector, it is widely utilized as an emulsifying agent, tablet binder, thickening or suspending agent [146]. Moreover, it is “Generally Recognized as Safe (GRAS)” according to the American Food and Drug Administration (USFDA) [147]. GA also contains salts such as Ca, Mg, and K, making it possible to utilize in microencapsulation, complex coacervation techniques, and as a drug delivery carrier [148]. Moreover, GA-based membranes can be synthesized by diverse techniques, making it suitable for implementation in many sectors, either in medical or environmental applications (Figure 11).

### 6.1. Applications of GA-Based Membranes or Scaffolds

#### 6.1.1. Tissue Regeneration

For scaffolding purposes, especially for bone regeneration applications, the significant amount of calcium in GA might help to decrease osteoporosis and improve treatment [149]. Moreover, if the polymeric organic matrix includes inorganic reinforcements such as these minerals, this will help in tissue regeneration applications as it will greatly resemble the real framework architecture of bone, and hence it can boost the biological and mechanical properties of the regenerated bone [150].

Based on the abovementioned information regarding GA, ternary nanosystem scaffolds comprising GA, κ-carrageenan (κ-CG), and nano-hydroxyapatite (n-HA) with different concentration ratios were fabricated via the facile co-precipitation method for bone tissue engineering purposes. The concentration ratios were 30/10/60, 20/20/60, and 10/30/60 for GA, κ-CG, and n-HA, respectively [150]. The results of scanning electron microscope visualization showed notable rapid mineralization and apatite layer formation after incubation for about 2 weeks in simulated body fluid (SBF) when compared to the binary system composed only of gum Arabic (GA) and nano-hydroxyapatite (n-HA) with a concentration ratio of 40/60. Moreover, a concentration ratio of 20/20/60 revealed the maximum apatite layer formation and good mechanical properties, which might be attributed to the presence of the identical amounts of GA and κ-CG in this scaffold. In addition, the scaffold containing the same amounts of GA and κ-CG exhibited good cytocompatibility and proliferation potential when cultured with osteoblast-like MG63 cells with good osteogenic differentiation in terms of the enhanced protein expression of osteonectin, osteopontin, and osteocalcin [150].

A GA film incorporating titanium oxide nanoparticles (TiO_2_ NPs) was fabricated using the solvent casting method, in which GA was first dissolved in deionized water, and glycerol and CaCl_2_ were subsequently added; then, TiO_2_ NPs were added while stirring and then sonicated at room temperature for 5 min. Afterward, the resultant solution was transferred to a casting dish to allow drying in an oven at 50 °C and film formation [151]. Morphological examination revealed the good dispersion of TiO_2_ NP filler within the GA film matrix, resulting in improved mechanical properties, biodegradability, and an excellent tendency to form a bone-like apatite layer. Moreover, the fabricated film showed excellent biocompatibility and good proliferation when incubated with MG-63 cells. In addition, it also exhibited a strong antibacterial effect against *Staphylococcus aureus* and *Escherichia coli*, confirming that this membrane scaffold might be a promising material for bone tissue regeneration materials [151].

GA and ε-polylysine composite films were immobilized via the layer-by-layer (LBL) assembly technique on anodized titanium with the aid of polydopamine [152]. The biomimetic polydopamine-functionalized surfaces of mussels can covalently graft molecules with amino groups in their structure [153], such as ε-Polylysine (ε-PL). ε-Polylysine (ε-PL) was included in this composite film as it is a typical antimicrobial peptide (AMP) in its structure, containing 25–30 lysine residues attached by α- carboxyl and ε- amino groups, and because it has been widely used as a food preservative agent [154]. Combining covalent bonding and LBL techniques, anodized titanium was first coated with polydopamine, then ε-PL was covalently grafted to coated anodized titanium. Afterward, the polyanion GA was introduced to assemble over ε-PL via electrostatic interaction, and this step was repeated to produce a multilayer composite film [152]. In vitro antibacterial investigations showed a decrease in the bacterial count when increasing the amount of ε-PL, with a long-term antibacterial effect against both Gram-negative and Gram-positive bacteria for multilayers of ε-PL and GA for up to 21 days. Interestingly, when TiO_2_ nanotube (TNT)-modified composite films were incubated with rat bone marrow mesenchymal stem cells (BMSCs), the cells exhibited excellent proliferation and osteogenic differentiation, suggesting that the coating of TNTs with ε-PL and GA increases their biocompatibility, which is an essential requirement for orthopedic implantation materials [152].

#### 6.1.2. Drug Delivery

GA is a negatively charged biopolymer that can be blended with positively charged polymers via ionic interaction, resulting in the formation of polyelectrolyte complex-based membranes or scaffolds [155]. Moreover, GA is usually added to reduce the viscosity of the solutions of some viscous polymers. GA is composed of a main galactan chain with branched arabinose/galactose side chains [156]. This branched architecture has fewer intermolecular interactions and a smaller hydraulic radius when compared to linear biopolymers, and hence GA solutions have very low viscosity [157]. In this light, chitosan/pectin/GA polyelectrolyte complex (PEC) membranes were fabricated with different ratios for the precise release of insulin as a model proteinaceous drug using a solvent casting technique [158]. Insulin was loaded into the membranes by adding chitosan-based solutions to allow the ionotropic complexation of the negatively charged insulin with the positively charged chitosan. The results indicated that the addition of GA to chitosan and pectin during preparation caused a significant decline in the zeta potentials and viscosities of the resultant PEC solutions, which might be attributed to the formation of globe-like microstructures and network-like microstructures. Moreover, the physicochemical characterization of the fabricated membranes showed an improvement in the hydrophilicity and the mechanical properties, especially for a weight ratio of 84/8/8 (chitosan/pectin/GA), compared to pure chitosan membranes. When the same membrane with weight ratios of 84/8/8 (chitosan/pectin/GA) was used as a drug carrier platform, it demonstrated steady and complete insulin release after 6 h, with the possibility of adjusting its release pattern by altering the amount of calcium utilized as a pectin cross-linker, confirming that these membranes can be tuned for controlled drug release applications [158].

#### 6.1.3. Antifouling Materials

Fouling is the deposition of unwanted materials on surfaces in close contact with water resources [159]. It is a natural phenomenon affecting a wide range of industrial sections such as heat exchange systems, cooling water towers, wastewater treatment, seawater desalination, and most importantly, drinking water systems due to all their components, including filtration membranes, storage tanks, distribution systems, and plumbing systems in buildings [160,161]. Generally, there are four different types of fouling related to water treatment membranes. The first and most common type is biofouling, caused by microorganisms or their extracellular polymers. The second type is colloidal fouling caused by humic particles, silt, or clay. The third type is organic fouling, caused by the deposition of lipids, proteins, or polysaccharides on the membranes. The fourth type is the mineral precipitation of relatively low water-soluble salts such as barium, calcium, or magnesium salts on membranes [162].

Unlike other types of fouling, biofouling remains the most challenging type as it is difficult to overcome or control since other fouling types can be controlled by treating the membranes with diverse physical or chemical pretreatments [163]. It is an important problem for membranes, especially those under pressure [164]. Biofouling occurs when microorganisms adhere, grow and produce their extracellular polymers onto the surface of membranes, leading to the production of a thin layer of microbial biofilm [165]. For biofouling to occur, there should be organic soluble foulant particles to serve as the organic matter source for bacterial colonization [166]. In addition, the production of bacterial extracellular polymers attracts more microorganisms to come and adhere via bacterial signaling through quorum sensing [167]. This bacterial accumulation on the membranes can cause a drastic decline in the water flux due to a decrease in the membrane permeability, which results in a higher operating pressure to compensate for this decline in the flux and, hence, more energy consumption. To overcome these consequences, frequent membrane replacement or cleaning is required [163].

Membrane cleaning can be performed physically via ultrasound, air sparging, or flushing. Chemical methods include utilizing acids, bases, surfactants, metal-chelating agents, or enzymes to kill microorganisms [168]. Unfortunately, microorganisms can easily regrow depending on the dead bacterial biomass used as support [169]. Another effective solution is to modify the surface of the membranes via polymer coating, blending, or grafting materials with antimicrobial effects, which might give membranes with antimicrobial properties [170]. It has been reported that GA extract can efficiently improve the properties of membranes, such as their charge, porosity, flux, hydrophilicity, and resistance to biofouling, due to its well-known antibacterial activity [171]. GA was found to efficiently reduce bacterial colonization on the surface of membranes, which might be related to the hydrophilicity of the GA molecule, which can provide the surface of the membranes with anti-adhesive properties, as a thin film of water molecules will attach to the surface of the membrane, preventing bacterial attachment and biofilm formation. In another manner, the presence of membranes with negatively charged surfaces can induce stronger repulsion forces between negatively charged bacterial cells and negatively charged surfaces [172].

Based on this, polysulfone membranes incorporating different loading ratios of GA were fabricated via the phase inversion method, in which GA was blended during preparation at ratios 0.1, 1, 3, 5, and 7 wt. % [172]. The results revealed that the addition of GA, with its amphiphilic properties and negative charges to polysulfone membranes, caused a change in the overall porosity, charge, and hydrophilicity of the membranes. Moreover, there was a significant decrease in the bacterial attachment on the surface of the membranes incorporating GA when compared to the polysulfone membrane alone. In addition, it was shown that the decrease in the bacterial attachment was dependent on the amount of loaded GA, with a maximum decline in the bacterial colonization of both Gram-positive and Gram-negative bacteria observed when 7 wt. %. of GA was loaded in the casting solution, suggesting that modified membranes might have strong biofouling resistance and be suitable for water treatment applications [172].

A polyethersulfone membrane was fabricated via the phase inversion technique with different loading ratios of graphene oxide (0.1–5 wt. %) and 1 wt. % GA as a pore-forming and nanofiller agent [173]. Graphene oxide flakes showed a high content of oxygen-containing groups, confirming a high ratio of graphite oxidation and increased hydrophilicity. The results revealed that the prepared membranes exhibited high hydrophilicity and a negative surface charge, associated with increased porosity and water flux. Moreover, when the fabricated membranes were examined as antifouling materials using bovine serum albumin solutions and as anti-biofouling materials using bacterial suspensions and real treated sewage effluent, they demonstrated an outstanding antibacterial effect against Gram-negative and Gram-positive bacteria, suggesting that the incorporation of graphene oxide along with GA can increase the anti-biofouling potential of membranes as a consequence of the improved hydrophilicity and increased negative charge on the surface [173].

A polyvinyl chloride (PVC) membrane was modified by a magnetite@GA (Fe_3_O_4_@GA) nanocomposite. First, magnetite@GA was fabricated using the simple co-precipitation method, and then the fabricated nanocomposite was added to the PVC casting solution. Afterward, naked and modified PVC membranes were synthesized via the non-solvent-induced phase separation technique [174]. The results revealed that the addition of Fe_3_O_4_@GA to the casting solutions of PVC at a concentration of 0.25 wt. % could efficiently modify PVC membranes, reaching a water flux of about 52 L m^−2^·h^−1^·bar^−1^. Moreover, the filtration results revealed that the magnetite@GA/PVC membrane could remove organic contaminates with high rejection rates for the rifampicin antibiotics Reactive Red-195 and Blue-19. In addition, the incorporation of Fe_3_O_4_@GA into the PVC membrane resulted in improved hydrophilicity, permeability, and antifouling properties [174].

Hybrid membranes fabricated from cross-linked polyvinyl alcohol (PVA) incorporating nanocrystalline cellulose (NCC)/GA conjugates and a PEO-PPO-PEO block copolymer were fabricated via a solution casting method [175]. First, NCC was prepared via the acid hydrolysis of microcrystalline cellulose. The PVA membrane was then cross-linked with Tetraethylorthosilicate (TEOS) to overcome the problem of its swelling in aqueous solutions due to its highly hydrophilic nature. Afterwards, the PEO-PPO-PEO block copolymer was incorporated into cross-linked membranes to ensure pore formation, in addition to tuning the diffusion rate across the membrane, thus improving the flux rate [176]. For GA, it was included in this preparation owing to its negative charges and potent antibacterial effect [177,178]. The performance results showed that this hybrid cross-linked membrane containing 0.1 wt. % of NCC can efficiently remove boron, with a selectivity reaching 92.4% and a flux rate of 21.3 L/m^2^h. More importantly, the incorporation of GA into this membrane resulted in an improved antibacterial effect on the membrane surface by preventing or minimizing bacterial attachment, suggesting that these hybrid membranes can be a good choice for the removal of hazardous materials like boron [175].

Another hybrid membrane was fabricated using the phase inversion technique from polyethersulfone (PES) mixed with oxidized multi-walled carbon nanotubes (OMWCNTs), along with the addition of an equal amount of zinc oxide nanoparticles (ZnO NPs) and GA [179]. The results indicated that the inclusion of ZnO NPs/GA in the membrane fabrication caused an increase in the hydrophilicity of the membrane, with a significant reduction in the amount of negative charge on the membrane surface; this was associated with a doubling of the flux values and improved porosity. In addition, this hybrid membrane exhibited an approximately 13% increase in the rejection rate upon exposure to BSA (1000 ppm) solution compared to the membrane without ZnO NPs/GA, confirming its good resistance to protein contamination [179].

To improve the filtration properties of polyphenylsulfone (PPSU) membranes, graphene sheets modified with GA were first fabricated via a facile green method; these were then added while preparing the membrane composite using the non-induced phase separation technique [180]. It is worth mentioning that naked graphene sheets were not incorporated directly into the casting solution of PPSU membranes as a filler during preparation, but they were first modified with GA to overcome the problem of graphene hydrophobicity as it is very difficult to mix it with hydrophilic polymer solutions. The results indicated that the incorporation of GA-modified graphene sheets at a concentration of up to 0.25 wt. % revealed improved hydrophilicity and good permeation properties when compared to PPSU membranes without modification, with a massive transformation in the morphology from a finger-like structure to dense structure. In addition, these membranes demonstrated good thermal stability and a significant decrease in their pore size, suggesting that this modified membrane could be a suitable candidate for ultrafiltration applications [180].

#### 6.1.4. Food Packaging

Chitosan (CS), GA, and polyvinyl alcohol (PVA) composite films incorporating black pepper essential oil (BPEO) or ginger essential oil (GEO) were prepared via a solvent casting method [28]. The BPEO-PVA/GA/CS composite film exhibited a hetero-structure with entrapped BPEO droplets, increased roughness, and more cavities without cracks, which might be attributed to BPEO’s hydrophobic nature. In contrast, the PVA/GA/CS composite film, including GEO, showed a rough surface with more coarseness due to oil droplet migration toward the film surface. Both BPEO- and GEO-incorporated PVA/GA/CS composite films revealed considerable resistance to breakage and enhanced flexibility compared to the PVA/GA/CS composite film, along with improved heat stability. Regarding the essential oil (EO) retention and release rates, the BPEO-PVA/GA/CS composite film demonstrated superior performance over the GEO-PVA/GA/CS composite film. Additionally, both types of EO-incorporated films significantly inhibited the in vitro growth of *E. coli*, *B. cereus*, *S. aureus*, and *S. typhimurium*. These findings highlight the potential use of these composite films as alternatives for food packaging and wound-dressing materials [28].

A GA-based adhesive membrane encapsulating cinnamon essential oil in the vapor phase was developed as an active food packaging material [181]. Water-soluble polysaccharides such as GA are a well-known and safe backbone for creating graft-copolymerized acrylate monomers to prepare adhesive membranes [182]. Adhesive membranes incorporating essential oils usually have dual functions. On one hand, they can protect the oil from being oxidized, and on the other hand, they can regulate their release [183]. First, GA (5% wt./v) was grafted with 87 mmol of butyl acrylate (BA) and 3.5 m mol of hydroxyethyl methacrylate (HEMA) to prepare a water-adhesive membrane; then, 4, 8, and 10% *v*/*v* cinnamon essential oil was encapsulated within the membrane. The results revealed that the prepared membrane was able to prolong the release of the essential oil for up to 2 days. In addition, the 8% *v*/*v* loaded essential oil membrane displayed superior antibacterial activity against both Gram-negative and Gram-positive bacteria. More importantly, when cheese samples were packed with the prepared self-stick membranes, their shelf-life increased from 3 to 8 weeks, and when the cheese samples were inoculated with Shiga toxin-producing *E. coli* O157:H7 and packed with the self-stick membrane, there was a significant reduction in the total bacterial count for all the loading percentages of cinnamon essential oil, suggesting the potential use of this adhesive membrane as an active food packaging material [181].

#### 6.1.5. Biosensing

Chemical sensors or biosensors are commonly used in medical diagnosis, the detection of some environmental pollutants, resource investigation, and industrial manufacturing [184,185]. Biosensors fabricated from different materials should fulfill essential requirements, including high sensitivity, high selectivity, good stability, a fast response, facile fabrication, and a low cost of production [186]. Biosensors are usually composed of an interconnected set of tools for the conversion of an analyte concentration into quantitative or semi-quantitative data [187]. Any typical biosensor comprise three main parts, including bioreceptors that can recognize and capture an analyte, an interface matrix where reactions can occur, and a physicochemical transducer that transforms the generated data into quantified signals, probably electronic signals. There might be a detector that can amplify the signal produced by the transducer before its processing and analysis by computer software [188].

Biosensors, depending on the electrical measurements, work by creating an interaction between electrical energy and a chemical reaction, mainly an oxidation/reduction reaction that can produce an electric current. The chemical reaction occurring between the immobilized biomaterial and the analyte results in either the production or consumption of ions or electrons, which in turn affect the electrical properties of the solution by affecting the electrical current or the electrical potential. These reactions usually occur at the interface between a metal or semiconductor electrode and an electrolyte [189]. As a result, the detection will be applicable if the reaction occurs in close contact with the electrode surface, and hence, it can be concluded that the electrode should be carefully selected as it strongly affects the performance of biosensors. Most biosensors, depending on the electrochemical signals in their detection, are composed of three electrodes, which are the reference electrode, the counter or auxiliary electrode, and the working electrode. The reference electrode is usually fabricated from Ag/AgCl, and it is used to normalize the readings. The counter electrode is the source of current applied to the working electrode. The working electrode itself acts as the transducer component in the reaction [189].

Screen-printed technology was recently used in the reference electrode miniaturization of Ag/AgCl. This technology changes the ordinary large-sized electrodes into thin and planar electrodes, causing an automatic change in the liquid electrode to a solid one. This screen-printed electrode has a simple shape, and it can be easily prepared in bulk [190]. This is compared with the vAg/AgCl reference electrode. In addition, the layers of Ag/AgCl can dissolve easily in the 3 M KCl electrolyte solution, causing the electrode to be unstable and have a very short lifetime [191]. In an attempt to improve the stability of the Ag/AgCl reference electrode, GA was utilized to fabricate a hydrogel membrane layer to coat the surface of the reference electrode [192]. This natural polymer was utilized due to its known inhibitory or decelerating effect on the corrosion rate of metals when immersed in acidic media [193]. The GA concentration utilized in this study ranged from 10 to 40% (*w*/*v*), with the optimum results obtained with two layers of GA membrane at a concentration of 20% (*w*/*v*). The efficiency of the electrode was examined according to the ∆ mV response of the Cl^–^ ion measurements, stability tests, and performance testing against ion-selective electrode (ISE) sensors such as NH_4_^+^, K^+^, and NO_3_^–^. The results indicated that the Ag/AgCl reference electrode coated with GA membrane demonstrated good performance and stability upon incubation for 72 h in 0.01 M KCl solution, with a drift of <0.6 mV/h [192].

A biosensor for the detection of Phenylalanine (Phe) was developed, with GA-based polyurethane membranes being utilized to modify the platinum electrode (Pt). First, the GA-based polyurethane membrane was fabricated using the solution polymerization technique, and then L-Phe-selective electrodes were fabricated via the simple coating of the Pt electrode with various thicknesses of the GA-based polyurethane membrane [194]. A GA-based polyurethane membrane was utilized to coat the Pt electrode, as this negatively charged membrane has improved adhesive and coating properties, with selective permeability and the accumulation of Phe molecules at the surface of the electrode and the elimination of other interfering molecules. The response of the modified electrode was examined by the differential pulse voltammetry technique, and the results revealed an R2 value of 0.9972 when the concentration ranged between 200 and 1000 μM of L-Phe, with a limit of detection and quantification of about 48.01 μM and 144.02 μM, respectively. Moreover, when this modified electrode was examined for its repeatability and sensitivity via ten consecutive measurements, it exhibited 98.8% stable results between the first and the tenth reading, confirming its potential application [194] (Table 7).

## 7. Conclusions and Future Perspectives

Gum Arabic (GA) is a natural polymer with a complex structure comprising polysaccharides and glycoproteins. It is a water-soluble and edible polymer, making it widely used in the field of food industry. In addition, it has strong emulsifying properties, so it can be used as a stabilizer in the fabrication of many types of nanoparticles, including metal nanoparticles, protein nanoparticles and liposomes. Moreover, it is an anionic polymer that can be used in the fabrication of hydrogels or nanofibers in combination with other polymers for use in many medical and pharmaceutical applications. Furthermore, it has strong antibacterial properties, so it can be used to coat membranes for antimicrobial or antifouling applications. It can be concluded that this sophisticated polymer can be included in the development of many biocompatible materials, and in the next few years, the global market of GA materials is expected to grow more and more.

## Figures and Tables

**Table 1:** Biochemical composition of *A. senegal* and *A. seyal* gums on dry basis (mean ± standard deviation) [2].

Component (mg/g)	*A. senegal*	*A. seyal*
Ash content	889.0 ± 0.27	893.0 ± 0.02
Galactose	35.8 ±1.20	36.9 ± 1.05
Arabinose	30.3 ± 2.50	47.6 ± 0.60
Rhamnose	15.5 ± 0.35	3.0 ± 0.30
Glucuronic acid	17.4 ± 1.15	6.7 ± 0.40
Proteins	27.0 ± 0.01	10.0 ± 0.04
Minerals	33.0 ± 0.24	40.0 ± 0.07

**Table 2:** Physical properties of Gum Arabic [4].

Property	Value
Gum Arabic Density	1.35–1.49 g/mL
Gum Arabic Molecular Weight	≈250 kDa
Gum Arabic Boiling Point	>250 °C
Gum Arabic Melting Point	0–100 °C

**Table 3:** Scopus database query.

Category	Limitation
Keywords used	“Gum Arabic” OR “Gum Arabic” OR “Acacia Gum”
Year of publication	2000–2024
Document type	Article
Publication stage	Final
Source type	Journal
Language	English

**Table 4:** Gum Arabic-based nanoparticles.

Formulation	Role of GA	Incorportaed Active Molecules	Preparation Method	Diameter	Application	Reference
GA-AgNPs	Reducing agent		Bio-reduction method	less than 10 nm	Potent anti *S. mutans* effect.	[34]
GA-AgNPs	Reducing agent		Bio-reduction method	220 nm	GA-AgNPs maintain their microbial activity after addition of toothpastes.	[35]
Ca (OH_2_)-loaded-CS/GA-NPs	Coating material	Calcium hydroxide	Polyelectrolyte complexation	60.47 nm	Improved anti-*E. faecalis* biofilm efficacy.	[36]
GA+CN-coated MF-loaded MSNs	Carbon source for bacterial enzyme Polymeric mesh for CN adsorption	Moxifloxacin	Modified Stöber method	190 nm	Elimination of more than 90% of the bacterial burden within infected bone in a rabbit osteomyletitis model.	[37]
ZnO nanofluids	Stabilizer	------	Precipitation method and microwave heating process	200–350 nm	Long-term storage stability for up to 6 months with enhanced antibacterial effect.	[38]
GA-capped CuNPs	Capping agent		Bio-reduction method	19.6–35.06 nm	Potent anti-*Salmonella typhimurium* activity. Photocatalytic action reduced 95% of crystal violet and methylene blue after 30 min.	[39]
CS/GA-coated liposomes	Coating agent	5I-1-indole	------	------	Potent antifungal action against the plant pathogen *Botrytis cinerea*.	[40]
MnO_2_-NPs	Reducing agent		Bio-reduction method	100 nm	Improved antiviral efficacy against influenza virus H1N1.	[32]
Carbon dots	Substrate for carbon dot production	Ciprofloxacin	Microwave-assisted pyrolysis of GA	~30 nm	Sustained release of the antibiotic with potent antibacterial action against both Gram-positive and Gram-negative bacteria.	[47]
GA-AuNPs	Coating agent		Chemical reduction	15–18 nm	PLNS-induced mice treated with GA-AuNPs with or without laser-activated extrinsic cancer apoptotic pathways.	[48]
GA-AuNPs	Coating agent		Chemical reduction	15–18 nm	Treatment of lung tumor-bearing mice with GA-AuNP/laser-activated intrinsic apoptotic pathways.	[49]
GA-AuNPs	Stabilizer		Chemical reduction	75–80 nm	Decreased HIF-1α and its regulator miRNAs and target gene c-Myc at 30% of the IC50.	[50]
Fe_3_O_4_ NPs@GA/AuNPs	Coating for Fe_3_O_4_ Reducing agent for AuNPs		Green reduction technique	20–35 nm	Anti-leukemia effect in a dose-dependent manner.	[51]
GA-Ga-NPs	Coating agent		Freeze-drying process	33–250 nm	GA-Ga-NPs displayed in vitro antioxidant, antihypertensive and antineoplastic properties.	[52]
NiO-NPs	Stabilizer		Sol–gel approach	59 nm	Photocatalytic activity with 50% growth inhibition of U87MG cells.	[53]
Spherical gold NPs (GNPs)-Carbon dots complex	Substrate for carbon dot production	Doxorubicin	Microwave-assisted heating andsucrose density gradient centrifugation	5–15 nm	Improved in vitro cytotoxicity against MCF-7 cell line.	[54]
Cur-loaded GA NPs	Wall material	Curcumin (Cur)	Self-assembly approach	457.4–470.1 nm	Resistant to UV radiation, with 82% of the drug remaining in the NPs after 5 h of exposure to UV.	[33]
Cur/GA/SA NPs	Wall material	Curcumin (Cur)	Ionotropic gelation method	10 to 190 nm	Potent antioxidant effect with high toxicity against HepG2 cells.	[55]
SBSA/GA/CMC nanocomplex	Stabilizer	Luteolin	Self-assembly technique	204 nm	High stability under varying pH and salt conditions.	[56]
Zein-GA-NPs	Stabilizer	EGC	Antisolvent precipitation technique	128.03 nm	Enhanced gastric stability, with only 16.42% of the drug released at pH 1.2.	[57]
β-Lg/GA nanocomplex	Wall material	EGC	Polyelectrolyte complexation	133 nm	Efficient antioxidant activity, associated with resistance to gastric acidity and good photostability.	[59]
CS/GA-NPs	Wall material	Quercetin	Ionic gelation process	267.3–493.2 nm	Enhanced antioxidant effect of quercetin in intestinal Caco-2 cell model due to improved cellular adhesion and permeation.	[60]
Zein-GA-tea polyphenols NPs	Stabilizer	Luteolin	Anti-solvent precipitation assay	202 nm	Excellent antioxidant effectiveness, with sustained release in SGF (26.4%).	[61]
Zein-GA-tea polyphenols NPs	Stabilizer	Luteolin	Anti-solvent precipitation assay	202 nm	Low calcium concentration promoted the physiochemical characteristics under various conditions.	[62]
CS/GA NPs	Wall material	Saffron extract	Ionic gelation approach	183–295 nm	Saffron release increased exponentially to 80% under acidic pH after 1 h compared to 70% of saffron at neutral pH after 4 h.	[63]
(GA, alginate, xanthan, and tragacanth)/soy phospholipid/cheese whey nanocomposite	Stabilizer	Gingerol	Green reduction method	~100 nm	Combintion of GA and tragacanth gum with whey liposomes resulted in fast wound healing and full closure after 72 h of treatment.	[64]
(GA or sodium alginate)/soy phosphatidyl-choline liposomes	Stabilizer	Curcumin	Ethanol injection technique	148 nm (GA-liposomes) 299 nm (SA-liposome)	Sodium alginate liposomes exhibited better drug retention and long-term storage.	[65]
AuNPs	Stabilizer and contrast agent		Pulsed laser ablation process	1.85–0.99 nm	Improved CT imaging with good brightness and image quality.	[66]
GA-Ag NPs	Stabilizer		Bio-reduction method	15–20 nm	Chemical sensor of H_2_O_2_ and glucose.	[67]
GA-Fe_3_O_4_ NPs	Coating agent		Bio-reduction method		Magnetic support for trypsin enzyme immobilization with the retention of activity up to 15 cycles under various conditions.	[68]

**Table 5:** Gum Arabic-based hydrogels.

Gel Composition	Preparation Method	Incorporated Active Molecules	Application	Reference
Chitosan, GA	High-pressure CO_2_		Tissue engineering.	[75]
Gelatin, GA aldehyde	Covalent cross-linking		Spheroid cell culture.	[76]
GA	Reverse micellization	Diethylenetriamine and taurine	Antibacterial hydrogel.	[78]
GA, chitosan, and nano hydroxyapatite	Physical cross-linking (FeCl_3_) Covalent cross-linking genipin		Bone regeneration.	[80]
Periodate oxidized GA and PVA	Covalent	Folic acid	Oral drug delivery.	[84]
Succinic-anhydride-modified chitosan, and mulialdehyde GA	Covalent	Nanocurcumin	Injectable hydrogel for locally accessible tumours.	[86]
Chitosan-GA and PVA	Freeze–thawing cycles	Ibuprofen	Transdermal drug delivery.	[87]
Pea protein and GA	Physical interactions	Thyme essential oil	Oral drug delivery.	[88]
GA-TG-Ag NPs and acrylamide	Covalent	Meropenem	Oral drug delivery.	[89]
GA and microcrystalline cellulose	Covalent	Sulfadiazine	Antimicrobial Application.	[90]
C13-tryptophan-tyrosine (C13-WY)) with GA	Physical interaction	Docetaxel	Ovarian carcinoma.	[91]
Polyaldehyde GA and carboxy methyl chitosan	Covalent	Doxorubicin	Cancer treatment.	[92]
Polyacrylic acid-co-Al^3+^-co-modified GA	Free radical polymerization and ionic interaction		Wound healing.	[95]
GA and pectin	Ionic interaction	Naringin	Wound healing.	[96]
collagen/GA	Physical interaction	Ketorolac	Wound healing.	[97]
alginate-GA	Physical interaction	Nerve growth factor and carnosine	Wound healing.	[98]
alginate-GA	Physical interaction	S-acetamidomethyl Cys20, 31-EGF peptide	Wound healing.	[99]
GA and gelatin	Covalent	Allantoin		[100]
Glycidyl-methacrylate-modified GA and methylene bisacrylamide	Free radical polymerization	Chitin nanowhiskers	Biomedical devices	[103]
PVA and GA	Covalent	Graphene nanoplatelets, activated carbon black, and reduced graphene oxide	Wearable medical devices.	[104]

**Table 6:** Gum Arabic-based nanofibers.

Embedded Active Molecule	NF Composition	Incorporation Method	Diameter	Application	Reference
Gum Arabic-coated gold nanoparticles (GA-AuNPs)	Polyvinyl-alcohol (PVA)/Gum Arabic (GA)	Blend electrospinning	227 nm	Biocompatible scaffold for in vitro cell growth and possible cancer therapy.	[123]
-	Zein, gum arabic (GA), Calen-dula officinalis (C. officinalis) extract, and poly (ε-caprolactone) (PCL)	Blend electrospinning	Average pore size > 9 μm	Skin tissue engineering with good in vitro cellular proliferation and attachment.	[124]
Curcumin	keratin, Gum Arabic (GA), and γ-polyglutamic acid (PGA).	Blend in coaxial spinning techniques	200–300 nm	Facilitates wound healing. Accelerates re-epithelialization process.	[125]
Silver nanoparticles (AgNPs)	Polycaprolacton (PCL)-coated GA/PVA NFs	Core/shell blend using electrospinning	150 to 250 nm	Effective antimicrobial effect against different bacterial strains. Good alternative for commercial wound dressing.	[126]
ZnO NPs in isopropyl myristate	Gum arabic (GA)/ Pullulan (PUL)	Blend using centrifugal spinning.	40 nm	Improved treatment for *Acne vulgaris*.	[132]
Tyrosol- (TYS) functionalized chitosan AuNPs	Polyvinyl-alcohol (PVA)/gum arabic (GA)	Core/shell blend using electrospinning	170 ± 38 nm	Inhibit fungal biofilm formation.	[138]
Geraniol with β-cyclodextrin	Polyvinyl-alcohol (PVA)/gum arabic (GA)	Inclusion using electrospinning	142 ± 61 nm	Eradicates fungal biofilms.	[140]
*Lactobacillus bulgaricus* (LB)	Gum Arabic (GA)/pullulan (PUL)/chia mucilage solution (CPS)	Blend electrospinning		Improved survival for probiotics for a longer time.	[145]

**Table 7:** Gum Arabic-based membranes or scaffolds.

Membrane, Scaffold or Film Composition	Preparation Method	Incorporated Molecule(s)	Application	References
Gum Arabic, κ-carrageenan, scaffold	Co-precipitation	Nano-hydroxyapatite (n-HA)	Bone tissue regeneration.	[150]
Gum Arabic, glycerol, CaCl_2,_ film	Solvent casting	TiO_2_ NPs	Bone tissue regeneration.	[151]
GA and ε-polylysine films immobilized on anodized titanium modified with polydopamine	Layer-by-layer assembly		Orthopedic application with potent antibacterial effect.	[152]
Chitosan/pectin/GA membrane, CaCl_2_	Ionotropic complexation and solvent casting	Insulin	Controlled drug release.	[158]
Polysulfone, GA	Phase inversion		Biofouling resistance.	[172]
Polyethersulfone, 1 wt. % GA	Phase inversion	Graphene oxide flakes (0.1–5 wt. %)	Fouling resistance.	[173]
Polyvinyl chloride (PVC) membrane	Non-solvent-induced phase separation	Magnetite@GA	Enhanced antifouling properties with the removal of organic contaminates.	[174]
Polyvinyl alcohol (PVA) membrane cross-linked with Tetraethylorthosilicate (TEOS)	Solvent casting	Nanocrystalline cellulose (NCC)/GA conjugate and PEO-PPO-PEO block copolymer	Efficient boron removal with good antifouling properties.	[175]
Polyethersulfone (PES), oxidized multi-walled carbon nanotubes (OMWCNTs),	Phase inversion	ZnO NPs, GA	Good resistance to protein contamination.	[179]
Polyphenylsulfone (PPSU) membranes	Non-induced phase separation	GA-modified graphene sheets	Ultrafiltration applications.	[180]
PVA/GA/Chitosan	Solvent casting	Black pepper essential oil (BPEO) and ginger essential oil (GEO)	Use as food packaging or wound-dressing material due to its potent in vitro antibacterial activity.	[28]
GA grafted with butyl acrylate and hydroxyethyl methacrylate adhesive membrane	Graft copolymerization	Cinnamon essential oil	Active food packaging material for increasing the shelf-life of cheese due to its good antibacterial activity.	[181]
Ag/Ag Cl reference electrode coated with two layers of GA membrane (20% *w*/*v*)	Solvent casting		Increased stability of the Ag/AgCl reference electrode in biosensors.	[192]
Platinum electrode (Pt) coated with GA-based polyurethane membranes then L-Phe selective	Solution polymerization technique		Improved molecules at the surface of the electrode and the elimination of other interfering molecules, associated with good sensitivity.	[194]

## Data Availability

Data will be made available upon request.
